# A migrasome-related lncRNA signature predicts prognosis and immune response in hepatocellular carcinoma: Implications for biomarker discovery and therapeutic targeting

**DOI:** 10.3389/fphar.2025.1581122

**Published:** 2025-08-06

**Authors:** Haotian Qin, Tiantian Qi, Min Liu, Weibei Sheng, Junyu Qian, Jian Weng, Qi Yang, Jun Yang

**Affiliations:** ^1^ Department of Bone & Joint Surgery, Peking University Shenzhen Hospital, Shenzhen Peking University-The Hong Kong University of Science and Technology Medical Center, Shenzhen, Guangdong, China; ^2^ National & Local Joint Engineering Research Center of Orthopaedic Biomaterials, Peking University Shenzhen Hospital, Shenzhen, China; ^3^ Emergency Department, Peking University Shenzhen Hospital, Shenzhen, China; ^4^ Department of Ultrasound Medicine, Peking University Shenzhen Hospital, Shenzhen, China; ^5^ Department of Radiology, Peking University Shenzhen Hospital, Shenzhen, China

**Keywords:** hepatocellular carcinoma, migrasome-related long non-coding RNAs, immune microenvironment, prognostic signature, immunotherapy

## Abstract

**Background:**

Hepatocellular carcinoma (HCC) remains a leading cause of cancer-related death, with limited response rates to immunotherapy. Identifying novel biomarkers to predict prognosis and guide treatment is urgently needed.

**Methods:**

Using TCGA-LIHC data, we identified migrasome-related long non-coding RNAs (MRlncRNAs) associated with HCC prognosis and constructed a two-lncRNA signature (LINC00839 and MIR4435-2HG) through LASSO-Cox regression. The model was validated in an independent cohort (n = 100). Multi-omics analyses were conducted to explore correlations with immune infiltration, immune checkpoints, TMB, MSI, and therapeutic sensitivity. Clinical sample validation and functional assays were performed to verify biological relevance. We knocked down MIR4435-2HG in HCC cells to assess its impact on proliferation, migration, EMT phenotype, and PD-L1 expression.

**Results:**

The MRlncRNA signature effectively stratified HCC patients by prognosis and immunotherapy responsiveness. High-risk patients exhibited elevated immunosuppressive cell infiltration and immune checkpoint expression. Functional validation revealed that MIR4435-2HG promotes malignant behaviors and immune evasion by regulating EMT and PD-L1. Single-cell analysis showed its enrichment in cancer-associated fibroblasts, suggesting a role in tumor-stroma crosstalk and immune suppression.

**Conclusion:**

MRlncRNAs, particularly MIR4435-2HG, contribute to HCC progression and an immunosuppressive tumor microenvironment. This study establishes a robust prognostic model and identifies potential targets for precision immunotherapy in HCC.

## Introduction

Hepatocellular carcinoma (HCC) is one of the most common and lethal malignancies worldwide, ranking as the sixth most prevalent cancer and the third leading cause of cancer-related deaths globally (Bray et al., 2018). The development of HCC is closely associated with chronic liver diseases, including hepatitis B and C virus infections, alcoholic liver disease, and non-alcoholic fatty liver disease, which induce persistent liver inflammation and fibrosis, ultimately leading to carcinogenesis (Gilles et al., 2022; Sung et al., 2021). Despite advances in diagnostic techniques, HCC is often asymptomatic in its early stages, and by the time clinical symptoms manifest, the disease is typically at an advanced stage with a high risk of local recurrence and distant metastasis. This poses significant challenges for early diagnosis and effective treatment (Yang et al., 2023). Current therapeutic options for HCC, including surgical resection, local therapies, and systemic chemotherapy, offer limited benefits for advanced-stage patients, highlighting the urgent need for novel targeted therapies and immunotherapeutic strategies to improve patient outcomes (Liu et al., 2023; Sadagopan and He, 2024).

In recent years, the discovery of migrasomes, a novel cellular structure formed during cell migration, has shed new light on tumor biology. Migrasomes are extracellular vesicles released by migrating cells, enriched with proteins and signaling molecules that play critical roles in regulating the tumor microenvironment (TME). These structures facilitate intercellular communication and influence tumor progression by promoting cancer cell invasion, metastasis, and drug resistance (Jiang et al., 2023; Deng et al., 2024). In HCC, migrasome-related genes (MRGs) have been implicated in tumor progression, where they enhance cell migration, invasion, and metastasis by modulating cell signaling and the dynamic interactions within the TME (Zhang K. et al., 2024). Furthermore, MRGs may also regulate immune cell infiltration, thereby influencing immune evasion mechanisms and contributing to tumor growth and metastasis (Zhang R. et al., 2024).

Long non-coding RNAs (lncRNAs), once considered “junk DNA,” have emerged as crucial regulators of gene expression, chromatin remodeling, and post-transcriptional modifications. They play pivotal roles in various cellular processes, including proliferation, migration, and invasion, and are increasingly recognized for their involvement in cancer progression (Gupta et al., 2010; Han et al., 2022; Li et al., 2023). In HCC, specific lncRNAs, such as HOTAIR and MALAT1, have been shown to drive tumor progression and metastasis (Kadian et al., 2024; Jia et al., 2024). Moreover, lncRNAs modulate the tumor immune microenvironment by influencing the function and distribution of immune cells, such as tumor-associated macrophages, thereby playing a role in immune evasion and response to immunotherapy (Tomczak et al., 2015; Yu et al., 2012). Given their regulatory roles, lncRNAs hold great promise as diagnostic, prognostic, and therapeutic biomarkers in HCC.

In this study, we systematically analyzed migrasome-related long non-coding RNAs (MRlncRNAs) in hepatocellular carcinoma (HCC) and developed a robust 2-lncRNA prognostic signature (LINC00839 and MIR4435-2HG) using TCGA data and LASSO–Cox regression. This signature was validated in clinical tissues and HCC cell lines, and was significantly associated with overall survival and immune-related features. Functional assays confirmed that MIR4435-2HG promotes proliferation, EMT, and PD-L1–mediated immune evasion. Moreover, MRlncRNA expression correlated with immune cell infiltration, immune checkpoint activation, and response to immune checkpoint inhibitors (ICIs), as evaluated by the TIDE algorithm.

Collectively, our findings reveal that MRlncRNAs contribute to HCC progression and immunosuppressive remodeling of the tumor microenvironment. This work identifies novel biomarkers for prognosis and immunotherapy prediction and offers mechanistic insights into the migrasome–lncRNA–immunity axis, paving the way for personalized therapeutic strategies in HCC.

## Materials and methods

### Data sources and preprocessing

Twelve migrasome-related genes (MRGs) were initially retrieved from the GeneCards database (https://www.genecards.org/) using the keyword *“migrasome”*. These genes were further filtered and confirmed based on prior published studies that experimentally validated their roles in migrasome biogenesis and function, including *TSPAN4*, *NDST1*, *CPQ*, and *ITGAV* (Supplementary Table S1). Gene expression profiles and corresponding clinical information for liver hepatocellular carcinoma (LIHC) patients were downloaded from The Cancer Genome Atlas (TCGA) database (https://portal.gdc.cancer.gov/) (Tomczak et al., 2015), including data from 372 LIHC tumors and 50 normal liver tissues. Clinical variables included age, sex, stage, grade, TNM classification, and overall survival (OS) time and status (Supplementary Table S2). Expression data were normalized to transcripts per million (TPM). All data preprocessing and visualization were conducted in R software (v4.4.1) using the “ggplot2” package. The Wilcoxon test was applied for comparisons of gene expression between tumor and normal samples.

### Identification of migrasome-related lncRNAs (MRlncRNAs)

To identify MRlncRNAs, we extracted lncRNA and mRNA expression profiles from the TCGA-LIHC dataset. Pearson correlation analysis was conducted between the expression of the 12 MRGs and all lncRNAs using the “limma” package. LncRNAs were considered migrasome-related if they met the threshold of |correlation coefficient| > 0.55 and P < 0.001. In total, 191 MRlncRNAs were identified based on this criterion. Co-expression relationships were visualized using the “ggplot2” and “ggalluvial” packages in R.

### Construction of prognostic model based on MRlncRNAs

Univariate Cox regression analysis was performed on the 191 MRlncRNAs to identify candidates significantly associated with overall survival (P < 0.05), yielding 16 prognosis-related MRlncRNAs. To further optimize the model and avoid overfitting, LASSO (Least Absolute Shrinkage and Selection Operator) Cox regression with 10-fold cross-validation was conducted and repeated 1000 times. This process ultimately selected two MRlncRNAs—LINC00839 and MIR4435-2HG—as the most stable predictors with the highest prognostic contribution and minimal AIC value. These two lncRNAs were used to construct the final risk model. All model-building procedures were performed using R packages including *survival*, *caret*, *glmnet*, and *timeROC*, and visualized with *ggplot2*.

### Construction and Validation of prognostic model

Risk scores for each patient were calculated using a multivariate Cox regression model based on the expression levels and coefficients of the prognostic MRlncRNAs, following the formula:

$$\text{Riskscore}=\sum i\text{ Coefficient }\left(\text{MRlncRNAsi}\right)\times \text{Expression }\left(\text{MRlncRNAsi}\right)$$

The TCGA-LIHC cohort was randomly divided into a training set and a testing set in a 1:1 ratio. Patients were then stratified into high-risk and low-risk groups using the median risk score as the cutoff. Kaplan–Meier survival analysis and time-dependent ROC curve analysis were performed to evaluate the model’s predictive ability, using the survival, survminer, and timeROC packages in R. To further validate the robustness and generalizability of the model, we applied the same risk score formula to an independent clinical tissue cohort (n = 100) collected from our institution. This external cohort was randomly split into validation set 1 (n = 50) and validation set 2 (n = 50). Patients in each validation set were classified into high- and low-risk groups based on the median risk score, and survival analyses were conducted accordingly. Univariate and multivariate Cox regression analyses were also performed to determine the independent prognostic value of the risk score, with results presented using forest plots (generated by the forestplot package). The predictive performance of the MRlncRNA-based model was further assessed by comparing 1-, 3-, and 5-year ROC AUCs across the TCGA and clinical validation cohorts. Additional subgroup analyses were performed based on clinical parameters such as age, gender, grade, and stage. The R packages rms, pec, and dplyr were utilized for these evaluations.

### Establishment and calibration of prognostic nomogram

Based on the results of multivariate Cox proportional hazards analysis, a nomogram was developed to predict the 1-, 3-, and 5-year overall survival. Calibration curves for 1-year, 3-year, and 5-year survival were plotted to assess the consistency between the predicted and observed outcomes. Furthermore, a C-index curve was generated to confirm the predictive accuracy of the nomogram. The R packages used for these analyses include survival, regplot, rms, and survcomp.

### Functional enrichment analysis

Differentially expressed genes (DEGs) between high- and low-risk groups were obtained using the Limma package in R. The adjusted P-values were used to control for false positives in the TCGA database analysis, with “Adjusted P < 0.05 and |log2FC| ≥ 1” set as the cutoff for selecting differentially expressed genes. The clusterProfiler package was used to analyze the Gene Ontology (GO) functional enrichment of DEGs and their pathways in the Kyoto Encyclopedia of Genes and Genomes (KEGG). The GO analysis focused on three aspects: Biological Process (BP), Cellular Component (CC), and Molecular Function (MF). Additionally, Gene Set Enrichment Analysis (GSEA) was used to identify potential biological pathways (http://software.broadinstitute.org/gsea/index.jsp) (Powers et al., 2018).

### Genetic variations

The cBioPortal database (http://www.cbioportal.org/) (Gao et al., 2013) provides visualization tools for cancer genomic data analysis. Using TCGA data, the genomic landscape of MRlncRNAs was explored, and their mutation frequency in LIHC was assessed. The impact of MRlncRNAs mutations on LIHC patient survival was also studied. Additionally, we analyzed the effect of MRlncRNAs mutations on clinical variables, including the Buffa Hypoxia Score, Winter Hypoxia Score, Aneuploidy Score, Ragnum Hypoxia Score, Fraction Genome Altered, Last Communication Contact from Initial Pathologic Diagnosis Date, and Neoplasm Histologic Grade.

### Immune cell infiltration analysis

The correlation between MRlncRNAs and immune cell infiltration was calculated using ssGSEA and CIBERSORT algorithms. Results were visualized using R software’s ggplot2 package. For immune scoring, the immunedeconv package in R and CIBERSORT algorithms (Newman et al., 2015) were used to compare the degree of immune cell infiltration between high- and low-risk groups via the Wilcoxon test. Additionally, single-sample gene set enrichment analysis (ssGSEA) (Hänzelmann et al., 2013) in the GSVA package [1.46.0] helped quantify the infiltration levels of immune cell types and the accumulation of 24 common immune cells. The Wilcoxon rank sum test was used to compare immune cell infiltration levels between high- and low-risk groups. The estimate package [1.0.13] in R was used to calculate immune cell abundance (immune score), stromal cell infiltration level (stromal score), and the combined score (ESTIMATE score).

### Immunotherapy response analysis

Risk scores and the correlation with immune checkpoint-related genes were visualized using the R packages “ggplot2” and “pheatmap”. Expression differences of immune checkpoint-related genes between high and low-risk score groups were analyzed, alongside the correlation between the expression of two prognostic MRlncRNAs and three clinically important immune checkpoint genes (CD274, CTLA4, PDCD1). Additionally, the prognostic significance of combining risk scores with these three immune checkpoints (CD274, CTLA4, PDCD1) in LIHC patients was assessed. Key immune checkpoint-related genes in this study included CD274, CTLA4, HAVCR2, LAG3, PDCD1, PDCD1LG2, TIGIT, and SIGLEC15. The Tumor Immune Dysfunction and Exclusion (TIDE) algorithm was used to predict potential immune checkpoint-blocking responses, with the results visualized using “ggplot2”. Distributions of TIDE data were compared between high and low-risk score groups. Finally, clinical tissue samples were used to predict the response of risk scores to immunotherapy.

### Single-cell sequencing data analysis

The Tumor Immune Single Cell Center (TISCH) (http://tisch.comp-genomics.org/) is a database for single-cell RNA sequencing (scRNA-seq) focused on the tumor microenvironment (TME) (Sun et al., 2021). The t-distributed stochastic neighborhood embedding (t-SNE) and heatmap of GSE125449 were generated using the TISCH database to explore the impact of MRlncRNA expression on the TME in LIHC. Furthermore, Spearman’s correlation method was applied to examine the relationship between MRlncRNAs and markers of cancer-associated fibroblasts (CAFs) and epithelial-mesenchymal transition (EMT).

### TMB, MSI, and potential drug screening analysis

To evaluate the clinical applicability of our risk model, we visualized the distribution of TMB and MSI scores across high and low-risk groups using stacked bar charts. Wilcoxon rank-sum tests were employed to compare TMB and MSI scores between these groups in the TCGA cohort, with results visualized using “ggplot2”. The “survminer” R package was used to calculate the optimal TMB cutoff, and patients with SARC in the TCGA cohort were classified into high and low TMB/MSI groups. Kaplan-Meier survival curves were used to assess prognostic differences. Additionally, drug sensitivity differences between high- and low-risk groups were analyzed using the limma, ggpubr, and pRRophetic R packages for prospective drug screening (Geeleher et al., 2014).

### Human sample collection

Tissue samples were provided by Peking University Shenzhen Hospital, including 100 pairs of liver cancer and adjacent non-tumor tissues, along with corresponding clinical and follow-up data. All patients underwent pathological diagnosis, and tissue samples were embedded in 10% formalin and stored under standard conditions. Archived samples collected between 2017 and 2019 and stored in the institutional biobank were used in this study. Comprehensive clinical information, including 5-year overall survival data, was retrieved from the hospital’s longitudinal medical records. The use of these archived samples for additional molecular validation experiments was approved by the Ethics Committee of Peking University Shenzhen Hospital (Approval No. 2024–116). Written informed consent was obtained from all patients prior to initial sample collection. All procedures complied with relevant ethical guidelines and institutional regulations.

### Cell culture conditions

The human normal hepatocyte cell line THLE-2 (CL-0833) was purchased from Procell Life Science & Technology Co., Ltd (Wuhan, China) and cultured in Procell’s specialized medium (CM-0833), a complete formulation designed for THLE-2 growth, containing basal medium supplemented with growth factors and hormones including epidermal growth factor (EGF) and insulin. Liver cancer cell lines HepG2, Huh7, and Hep3B were obtained from the American Type Culture Collection (ATCC, Manassas, VA, USA) and maintained in RPMI-1640 medium (Gibco) supplemented with 10% fetal bovine serum (FBS; Gibco) and 1% penicillin-streptomycin. All cells were incubated at 37 °C with 5% CO_2_ in a humidified atmosphere.

### RNA extraction and RT-qPCR

Total RNA was extracted using the Quick-RNA MiniPrep Kit (Zymo Research, Catalog No. R1054) according to the manufacturer’s instructions. RNA concentration and purity were assessed spectrophotometrically using a NanoDrop 2000 (Thermo Fisher Scientific). Reverse transcription was performed with 1 μg of total RNA using the miScript SYBR Green PCR Kit (Qiagen, Germany) on a LightCycler 96 real-time PCR system (Roche Diagnostics GmbH, Mannheim, Germany). PCR conditions were as follows: 95°C for 10 min, followed by 40 cycles of 95°C for 15 s and 60°C for 1 min. Gene expression levels were normalized to GAPDH and calculated using the 2-^△△^CT method. Primer sequences are listed in Table 1.

**TABLE 1:** The primers sequences utilized in RT-qPCR.

Real-time quantitative PCR primer sequence	
Gene	Sequence (5′- 3′ on minus strand)
*GAPDH*	Fwd: GAG​TCC​ACT​GGC​GTC​TTC​AC
	Rev: ATGACGAACATGGGGGCA
*LINC00839*	Fwd: GAA​CCT​GTG​GCA​TCC​ATC​TC
	Rev: CTC​CAG​CAA​CCC​CTC​AAC​C
*MIR4435-2HG*	Fwd: CGG​AGC​ATG​GAA​CTC​GAC​AG
	Rev: CAA​GTC​TCA​CAC​ATC​CGG​GC

### Cell transfection and knockdown of MIR4435-2HG

Short hairpin RNAs targeting MIR4435-2HG (sh-MIR4435-2HG) and corresponding negative control (sh-NC) were designed and synthesized by GeneRulor Biotechnology Co., Ltd. (Zhuhai, China). HepG2 and Huh7 cells were transfected using Lipofectamine™ 3000 (Invitrogen, USA) according to the manufacturer’s protocol. After 48 h, knockdown efficiency was evaluated by quantitative real-time PCR (qRT-PCR), and the most efficient sequence (sh-MIR4435-2HG#2) was selected for subsequent functional experiments.

### Cell proliferation assay

Cell proliferation was assessed using the Cell Counting Kit-8 (CCK-8) (Dojindo, Japan). Transfected cells were seeded in 96-well plates (3 × 10^3^ cells/well) and incubated for 0, 24, 48, and 72 h. At each time point, 10 μL of CCK-8 solution was added per well, followed by 2 h incubation at 37 °C. Absorbance at 450 nm was measured using a microplate reader (Bio-Rad, USA).

### Wound healing assay

After transfection, cells were grown to near confluence in 6-well plates, and a scratch was made using a 200 μL pipette tip. Detached cells were removed with PBS, and serum-free medium was added. Wound closure was imaged at 0 h and 24 h using a phase-contrast microscope. Migration rate was analyzed using ImageJ.

### Transwell migration and invasion assays

Cell migration and invasion abilities were evaluated using Transwell chambers (8 μm pore size, Corning, USA). For migration assays, 2 × 10^4^ cells in serum-free medium were seeded in the upper chamber. For invasion assays, chambers were precoated with Matrigel (BD Biosciences), and 5 × 10^4^ cells were seeded. The lower chamber contained 10% FBS as a chemoattractant. After 24 h incubation, cells on the upper membrane were removed, and migrated or invaded cells were fixed with 4% paraformaldehyde, stained with 0.1% crystal violet, and counted under a microscope.

### Western blot analysis

Total protein was extracted using RIPA buffer supplemented with protease inhibitors, and concentrations were determined using the BCA assay. Equal amounts of protein (30 μg) were separated by SDS-PAGE and transferred to PVDF membranes (Millipore, USA). After blocking with 5% BSA, membranes were incubated overnight at 4 °C with primary antibodies: Anti-E-Cadherin (ab40772, Abcam, United Kingdom, 1:1000), Anti-Vimentin (ab92547, Abcam, United Kingdom, 1:1000), Anti-PD-L1 recombinant antibody (ab205921, Abcam, United Kingdom, 1:1000), and Anti-GAPDH (ab8245, Abcam, 1:5000) as an internal control. After washing, membranes were incubated with HRP-conjugated secondary antibodies (1:5000, Abcam) for 1 h at room temperature. Protein bands were visualized using an ECL chemiluminescence substrate (Thermo Fisher Scientific) and imaged with a Tanon 5200 Multi chemiluminescence imaging system. Band intensities were quantified using ImageJ software and normalized to GAPDH.

### Statistical analysis

All statistical analyses were conducted using R (v4.2.1) (https://www.r-project.org/). The Student’s t-test was applied for normally distributed data, while the Wilcoxon test was used for non-normally distributed variables. Spearman’s correlation analysis was conducted to explore the relationships between variables. Chi-square or Fisher’s exact test was used for clinical feature analysis. Kaplan-Meier survival analysis, along with univariate and multivariate Cox regression analyses, were employed for prognostic assessment. P-values <0.05 were considered statistically significant (*p < 0.05, **p < 0.01, ***p < 0.001). All sections of the study were analyzed using specific datasets, R packages, and databases.

## Results

### Identification of Prognostic MRlncRNAs and Construction of Prognostic Features

**FIGURE 1:**
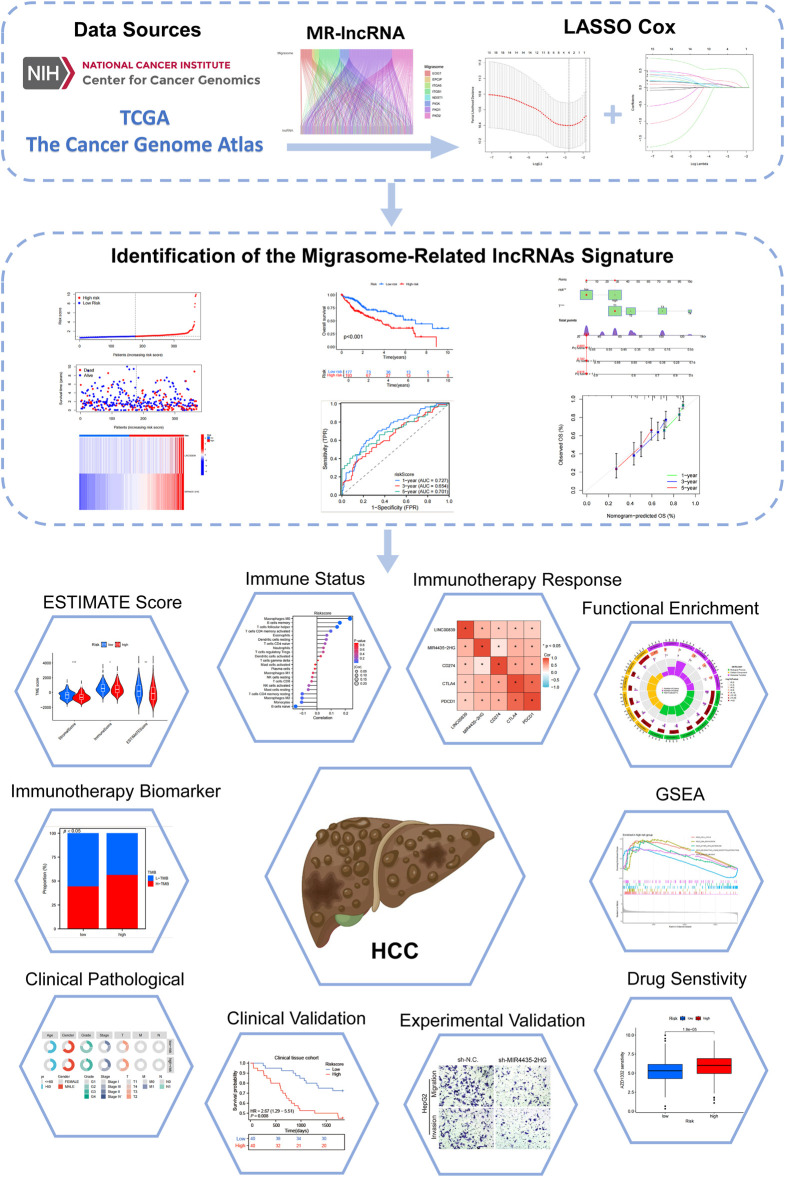
Flowchart of the present study.

**FIGURE 2:**
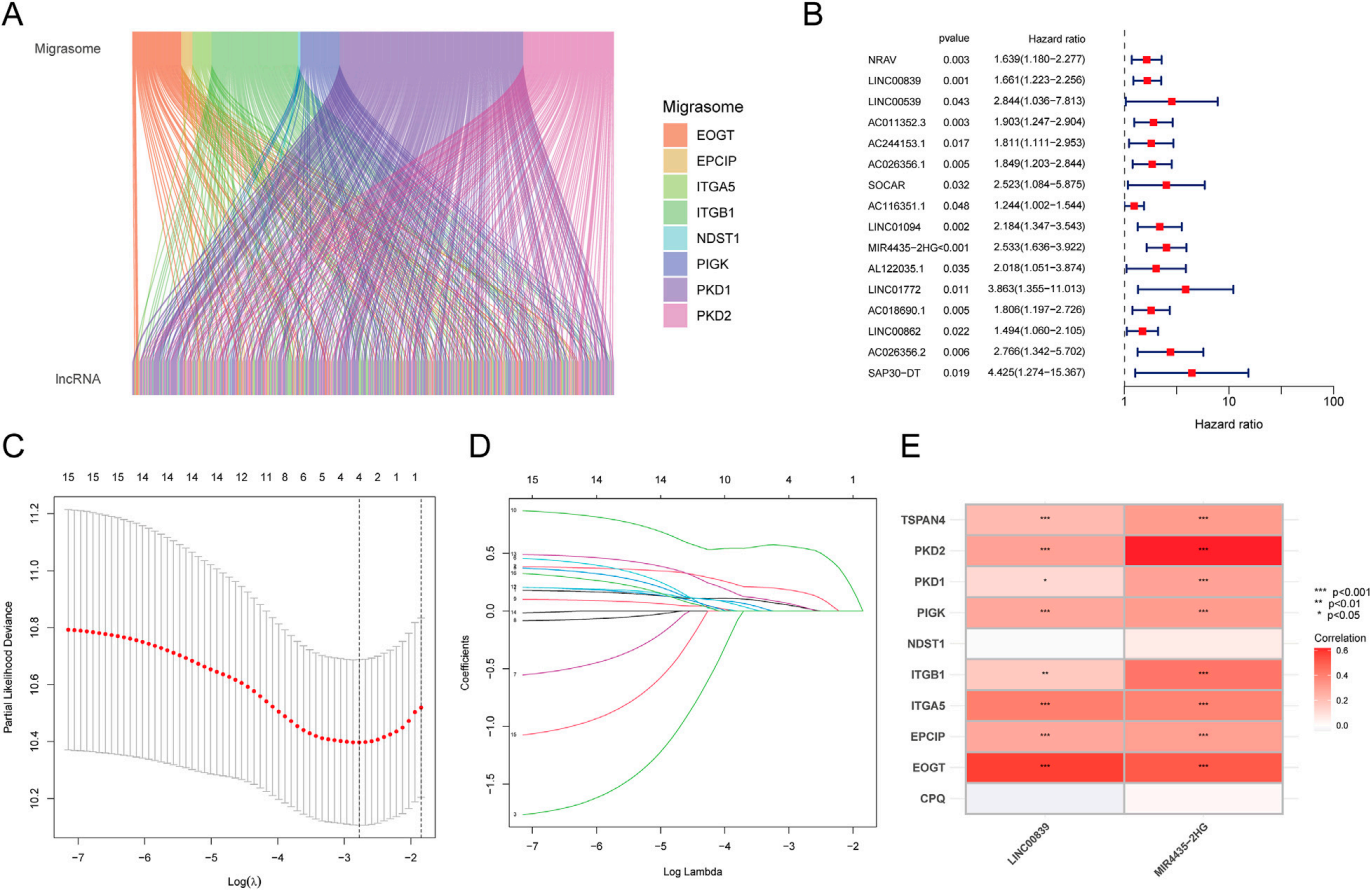
Identification of Prognostic MRlncRNAs and Construction of Prognostic Features. **(A)** Co-expression analysis of MRGs and lncRNAs; **(B)** Prognostic value of MRlncRNAs based on univariate Cox regression analysis (P < 0.05); **(C)** LASSO coefficient plot of prognostic MRlncRNAs; **(D)** Plot of the 10-fold cross-validation error rate; **(E)** Heatmap displaying the correlation between MRlncRNAs and MRGs.

The study flowchart is depicted in Figure 1. Expression levels of 12 MRGs were analyzed in 372 LIHC patients and 50 normal liver tissue samples from the TCGA-LIHC dataset. Co-expression analysis identified 191 lncRNAs significantly associated with migrasomes (Supplementary Table S3). Univariate Cox regression analysis revealed 16 MRG-lncRNAs significantly correlated with the survival prognosis of LIHC patients (P < 0.05) (Figure 2B). A prognostic model was developed using LASSO Cox regression based on these MRlncRNAs (Figures 2C,D). Multivariate Cox regression analysis further assessed the correlation of two MRlncRNAs, resulting in a risk score formula: RiskScore = LINC00839 × 0.299+MIR4435-2HG×0.818.

### Expression and prognostic value of MRlncRNAs

**FIGURE 3:**
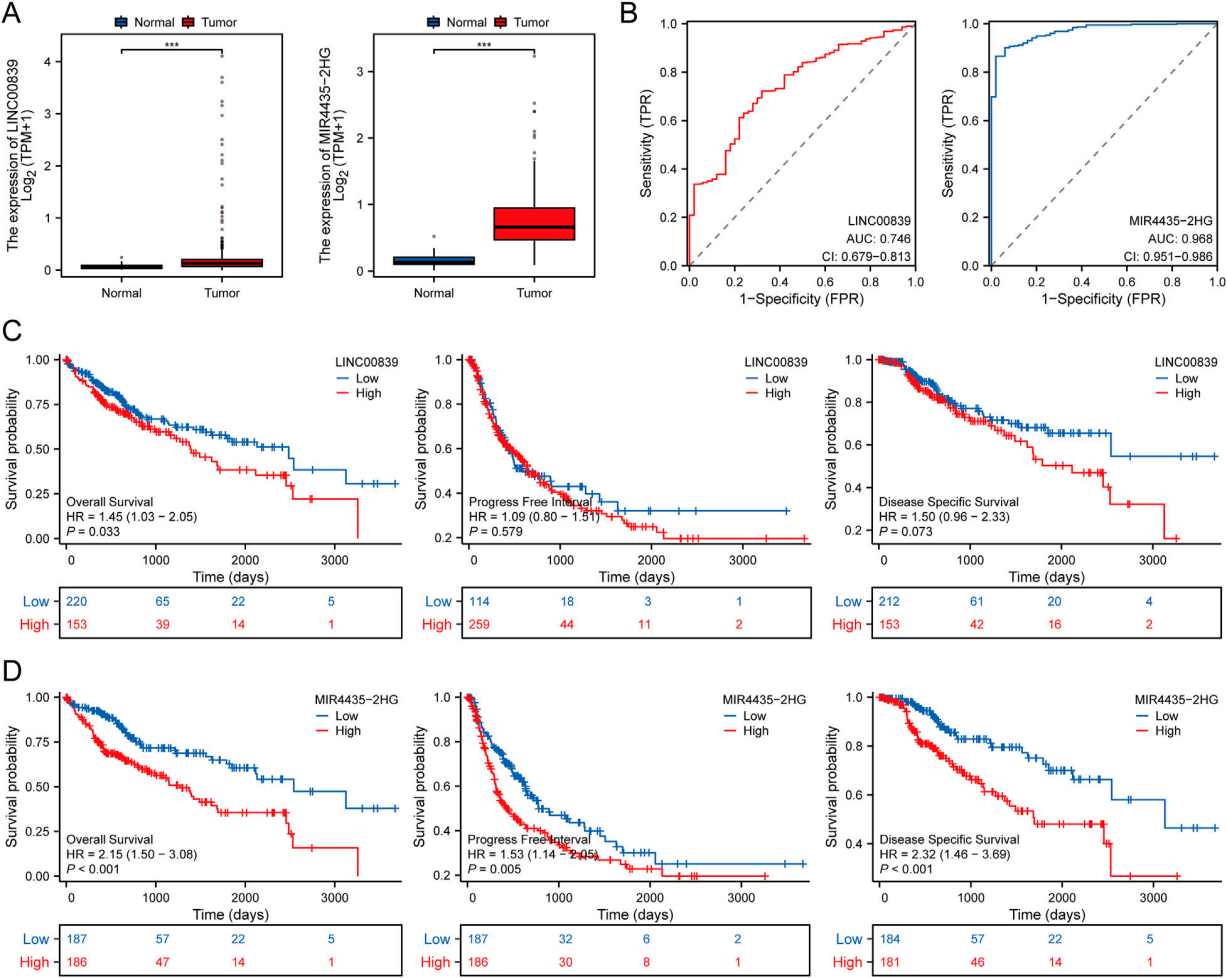
Expression and Prognostic Value Analysis of Prognostic MRlncRNAs in LIHC Patients. **(A)** Comparison of prognostic MRlncRNAs expression in liver tumors and normal tissues in TCGA databases; **(B)** ROC curves assessing the diagnostic potential of MRlncRNAs expression in LIHC; **(C)** Survival curves of OS, PFI, and DSS comparing high and low expression of LINC00839 in LIHC; **(D)** Survival curves of OS, PFI, and DSS comparing high and low expression of MIR4435-2HG in LIHC. *P < 0.05; **P < 0.01; ***P < 0.001.

The expression and prognostic performance of candidate MRlncRNAs were further evaluated within the TCGA discovery cohort. Compared to normal tissues, LINC00839 and MIR4435-2HG were significantly upregulated in liver cancer tissues (Figure 3A). ROC curve analysis showed that both lncRNAs had high diagnostic accuracy, with AUC values exceeding 0.7 (Figure 3B). Kaplan-Meier survival curves demonstrated that high LINC00839 expression was associated with poorer overall survival (OS; P = 0.033, HR = 1.45 [1.03–2.05]) and disease-specific survival (DSS; P = 0.073, HR = 1.50 [0.96–2.33]) (Figure 3C). Conversely, high MIR4435-2HG expression correlated with significantly worse OS (P < 0.001, HR = 2.15 [1.50–3.08]) and progression-free interval (PFI; P = 0.005, HR = 1.53 [1.14–2.05]) (Figure 3D). These findings suggest that MRlncRNAs are promising biomarkers for LIHC diagnosis and prognosis. Univariate and multivariate Cox regression analyses further identified MIR4435-2HG as an independent prognostic factor for LIHC (Table 2).

**TABLE 2:** Cox regression analysis of MRlncRNAs in prognosis prediction.

Characteristics	Total (N)	Univariate analysis		Multivariate analysis	
		Hazard ratio (95% CI)	P Value	Hazard ratio (95% CI)	P Value
Age	373				
≤ 60	177	Reference			
>60	196	1.205 (0.850–1.708)	0.295		
Gender	373				
Female	121	Reference			
Male	252	0.793 (0.557–1.130)	0.200		
Histologic grade	368				
G1&G2	233	Reference			
G3&G4	135	1.091 (0.761–1.564)	0.636		
Pathologic stage	349				
Stage I&Stage II	259	Reference		Reference	
Stage III&Stage IV	90	2.504 (1.727–3.631)	**< 0.001**	1.299 (0.177–9.518)	0.797
Pathologic T stage	370				
T1&T2	277	Reference		Reference	
T3&T4	93	2.598 (1.826–3.697)	**< 0.001**	2.241 (0.304–16.546)	0.429
Pathologic N stage	258				
N0	254	Reference			
N1	4	2.029 (0.497–8.281)	0.324		
Pathologic M stage	272				
M0	268	Reference		Reference	
M1	4	4.077 (1.281–12.973)	**0.017**	1.965 (0.602–6.412)	0.263
LINC00839	373				
Low	187	Reference			
High	186	1.306 (0.923–1.848)	0.132		
MIR4435-2HG	373				
Low	187	Reference		Reference	
High	186	2.147 (1.497–3.079)	**< 0.001**	2.189 (1.395–3.433)	**< 0.001**

### Establishment and validation of the prognostic MRlncRNA model

**FIGURE 4:**
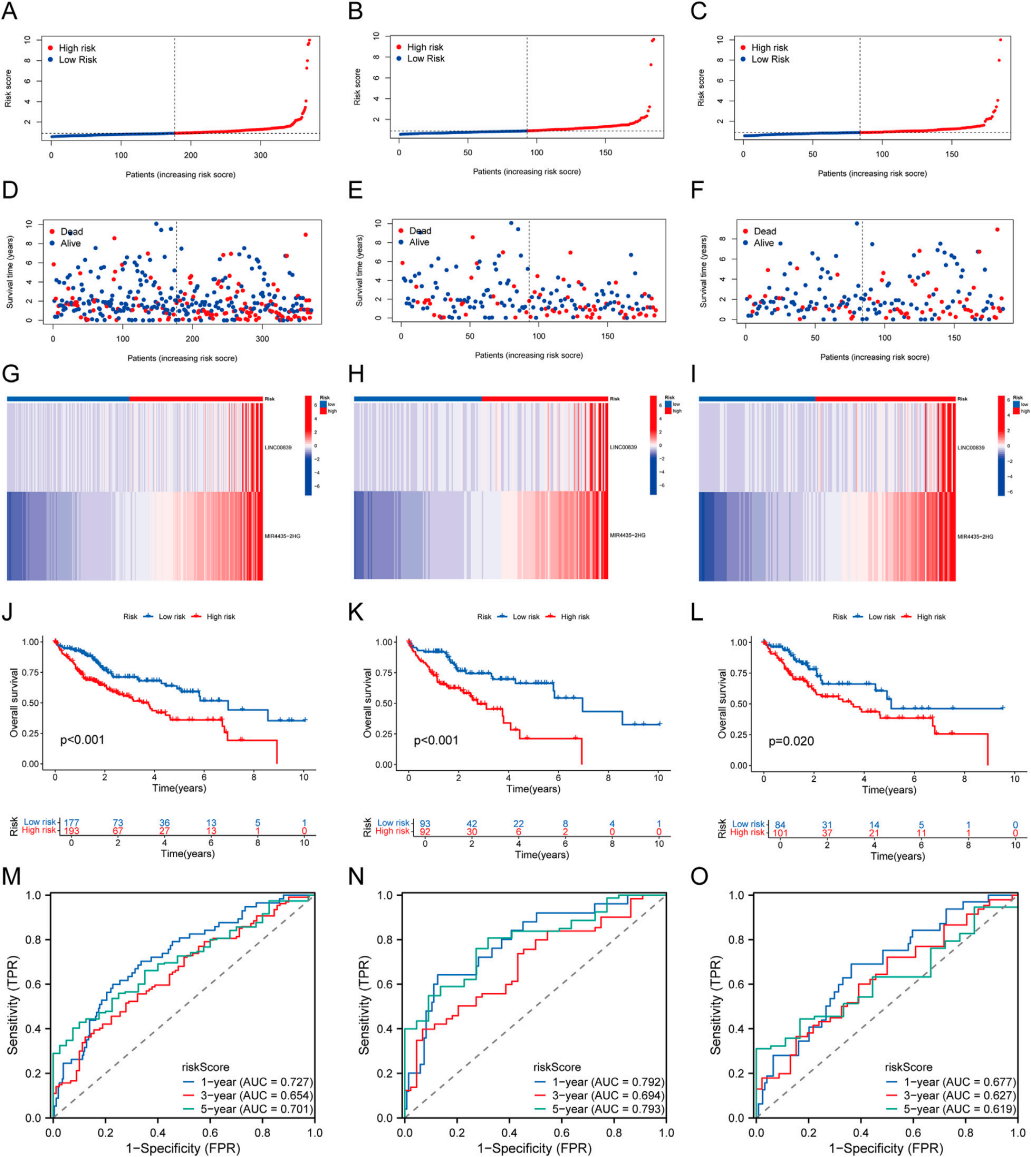
Establishment and Validation of Prognostic MRlncRNAs Features. **(A–C)** Distribution of risk scores for each patient in the TCGA-LIHC cohort, training cohort, and test cohort; **(D–F)** Distribution of overall survival status for each patient in the TCGA-LIHC cohort, training cohort, and test cohort; **(G–I)** Heatmap showing the expression of two prognostic MRlncRNAs in the TCGA-LIHC cohort, training cohort, and test cohort; **(J–L)** Kaplan-Meier survival curves for high-risk and low-risk groups comparing overall survival in the TCGA-LIHC cohort, training cohort, and test cohort; **(M–O)** Time-dependent ROC curves for 1-, 3-, and 5-year OS in the TCGA-LIHC cohort, training cohort, and test cohort.

The 370 TCGA-LIHC samples were randomly divided into training and validation sets. Patients were categorized into high-risk and low-risk groups based on median risk scores. As risk scores increased, mortality risk also increased, and survival time decreased (Figures 4A–F). Heatmaps illustrated the differential expression patterns of lncRNAs between risk groups (Figures 4G–I). Kaplan-Meier curves showed that high-risk patients exhibited significantly lower OS across all sample groups (P < 0.05, Figures 4J–L). The AUCs for 1-, 3-, and 5-year OS predictions in the entire cohort were 0.727, 0.654, and 0.701, respectively (Figure 4M). Similar results were observed in the training set (Figure 4N) and test set (Figure 4O), validating the predictive accuracy of our risk model.

### Correlation with clinicopathological features

**FIGURE 5:**
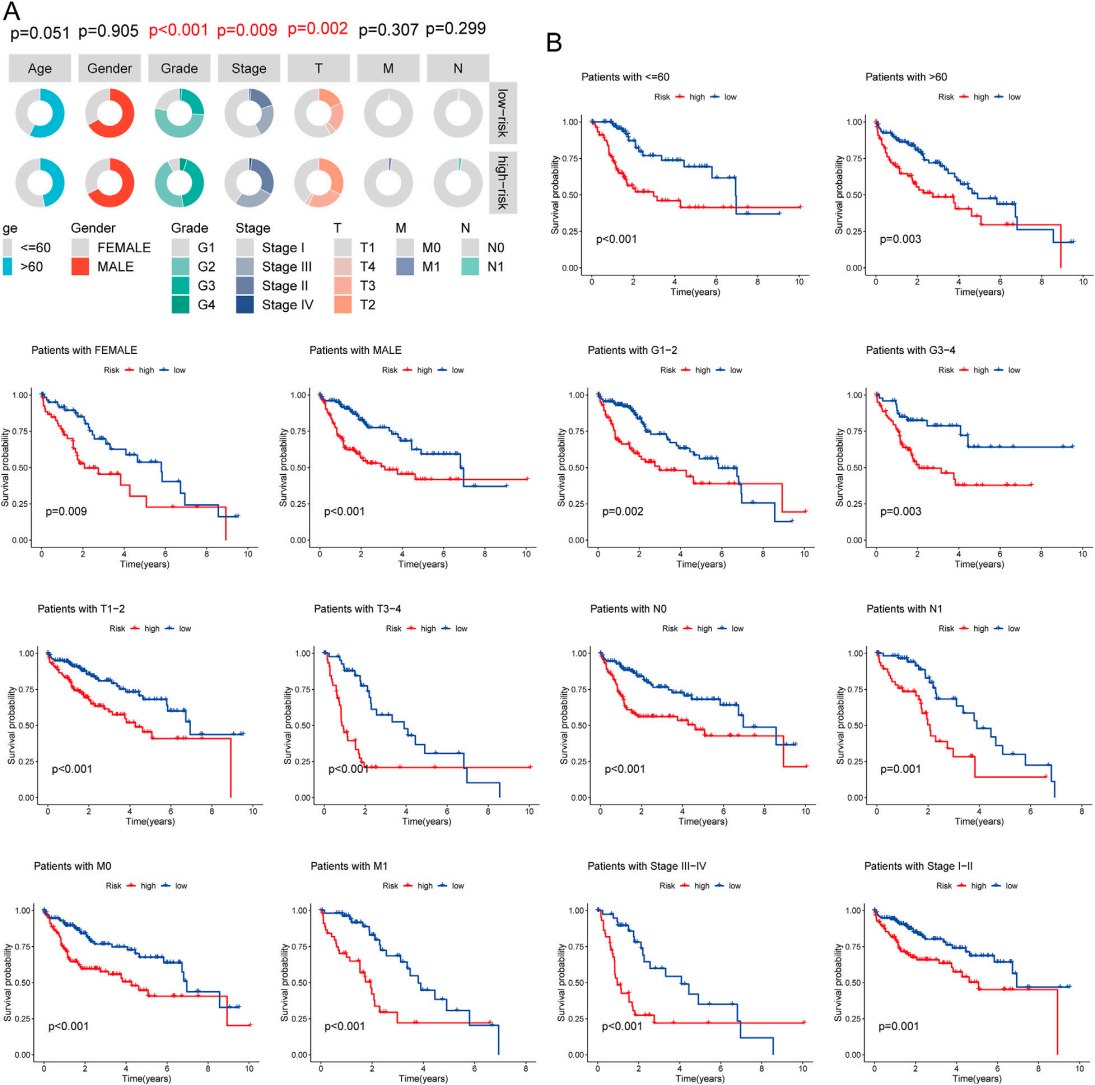
Association Between Risk Score and Clinicopathological Characteristics. **(A)** Circos plot showing the clinical factors between high and low-risk score groups; **(B)** Survival curve comparing high and low-risk score groups in different LIHC subgroups.

The association between risk scores and clinicopathological features was analyzed in the TCGA-LIHC cohort. Significant correlations were observed between risk scores and tumor grade, stage, and T status (Figure 5A; Supplementary Table S4). Subgroup survival analysis revealed that the high-risk group had significantly poorer survival across various clinical subgroups, suggesting that these factors play a critical role in LIHC prognosis (Figure 5B).

### Development and validation of a prognostic nomogram

**FIGURE 6:**
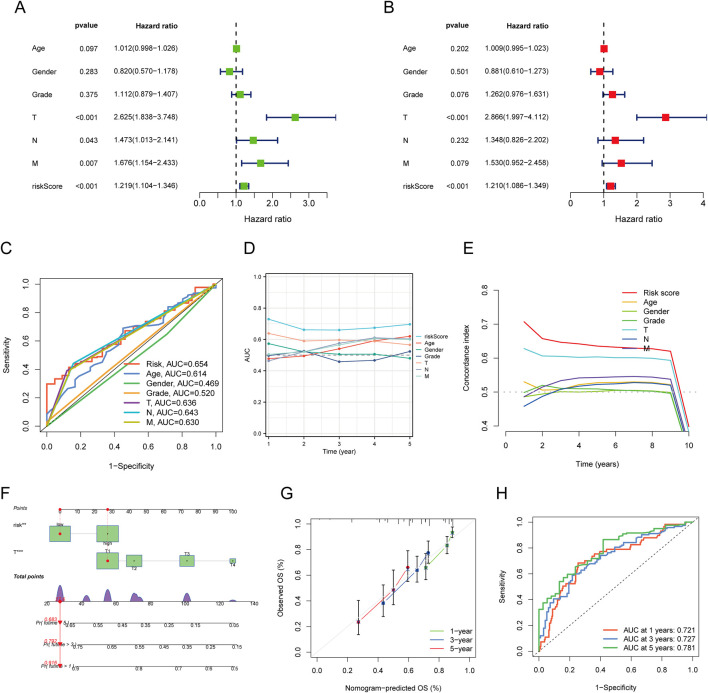
Construction and Validation of a Predictive Nomogram. **(A,B)** Univariate and multivariate Cox regression analysis of clinical variables in LIHC; **(C)** ROC curves for risk scores and various clinical pathological parameters (age, gender, Grade, T, N, M); **(D)** Time-dependent AUC curve showing the risk score’s performance in predicting OS; **(E)** Consistency index of the risk score and other clinical pathological variables; **(F)** Nomogram for predicting 1-, 3-, and 5-year OS of LIHC patients; **(G)** Calibration curve of the OS nomogram model in the discovery group, with the diagonal dotted line representing the ideal nomogram; **(H)** ROC curves for predicting 1-, 3-, and 5-year OS.

Univariate and multivariate Cox regression analyses identified T status (HR = 2.625, P < 0.001), N status (HR = 1.473, P = 0.043), M status (HR = 1.676, P = 0.007), and risk score (HR = 1.219, P < 0.001) as prognostic factors for OS in LIHC. The nomogram integrating risk score and T status was constructed to predict 1-, 3-, and 5-year OS in LIHC patients. ROC analysis of the nomogram demonstrated good predictive accuracy, with AUC values of 0.636 for the risk score model and 0.654 for the T status model (Figure 6C). The nomogram’s calibration curve confirmed a strong concordance between predicted and observed OS (Figure 6G), with AUCs of 0.721, 0.727, and 0.781 for 1-, 3-, and 5-year OS predictions, respectively (Figure 6H).

### Functional enrichment analysis of high- and low-risk MRlncRNAs

**FIGURE 7:**
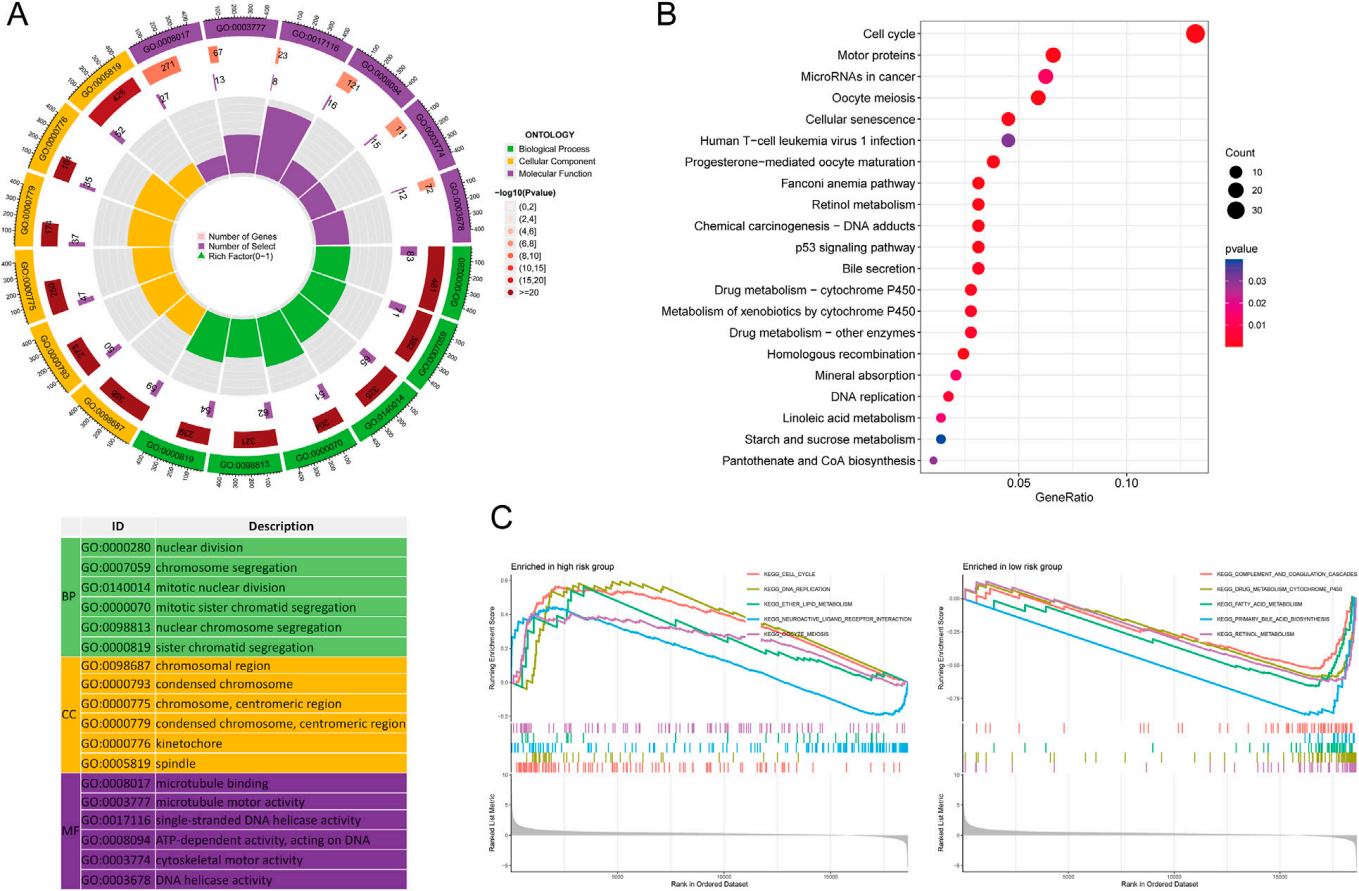
Functional Enrichment Analysis Between High and Low-Risk Groups. **(A)** GO enrichment results; **(B)** KEGG pathways; **(C)** GSEA analysis.

A total of 755 DEGs were identified between high- and low-risk MRlncRNA groups. GO analysis indicated that these DEGs were enriched in biological processes such as nuclear division, chromosome segregation, and mitotic division (Figure 7A). Cellular components included chromosomal regions and kinetochores, while molecular functions were enriched in microtubule binding and ATP-dependent DNA helicase activity. KEGG pathways were mainly associated with the cell cycle, microRNAs in cancer, and p53 signaling (Figure 7B). GSEA showed that genes in the high-risk group were primarily involved in the cell cycle, DNA replication, and neuroactive ligand-receptor interactions, while genes in the low-risk group were enriched in complement and coagulation cascades, fatty acid metabolism, and bile acid biosynthesis (Figure 7C).

### Genetic alteration analysis of MRlncRNAs

**FIGURE 8:**
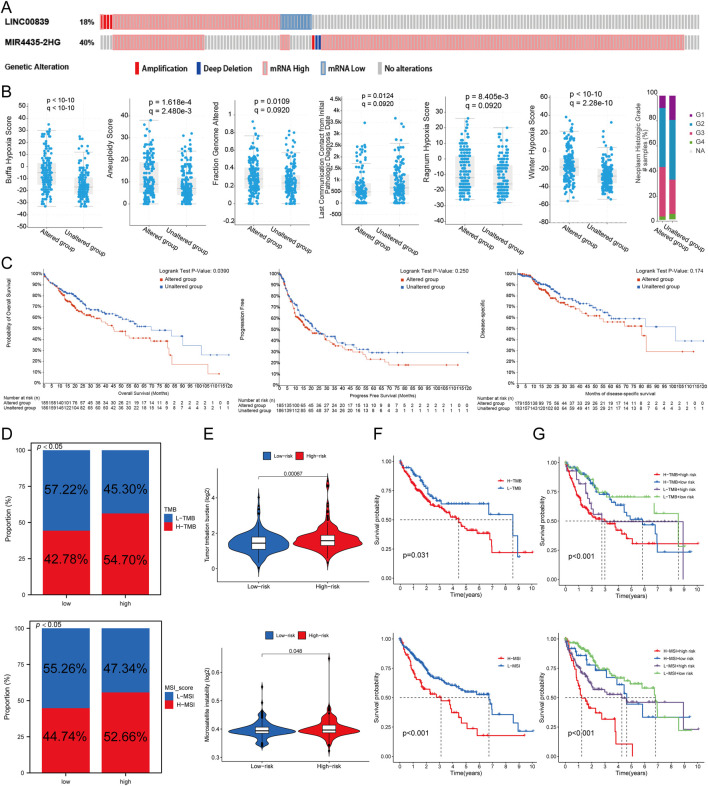
Genetic Alterations Associated with MRlncRNAs. **(A)** Oncoprint displaying somatic mutations in the LIHC tumor cohort; **(B)** Correlation between MRlncRNA alterations and various clinicopathological factors in LIHC, including Buffa Hypoxia Score, Winter Hypoxia Score, Aneuploidy Score, Ragnum Hypoxia Score, Fraction Genome Altered, Neoplasm Histologic Grade, and Last Communication Contact from Initial Pathologic Diagnosis Date; **(C)** Genetic alterations of MRlncRNAs in LIHC tissues correlated with shorter OS, PFS, and DFS in patients; **(D)** Distribution of TMB and MSI scores in high-expression and low-expression groups; **(E)** Differences in TMB and MSI between high and low-risk score groups in LIHC; **(F)** Kaplan-Meier survival curves for high and low TMB, MSI groups in LIHC; **(G)** Kaplan-Meier survival curves of four groups classified by risk score and TMB, MSI in LIHC.

Genetic alterations of MRlncRNAs were analyzed using the cBioPortal database. Mutations in LINC00839 and MIR4435-2HG were detected in 175 of 348 sequenced LIHC samples (Figure 8A). Clinical analysis revealed significant differences between the mutation and non-mutation groups in Buffa Hypoxia Score, Winter Hypoxia Score, Aneuploidy Score, Ragnum Hypoxia Score, Fraction Genome Altered, Last Communication Contact, and Neoplasm Histologic Grade (Figure 8B). Survival analysis showed that genetic alterations of prognostic MRlncRNAs were significantly associated with shorter overall survival (OS, P = 0.0390), but not with progression-free survival (PFS, P = 0.250) or disease-free survival (DFS, P = 0.174) (Figure 8C). Compared to low-expression patients, high-expression patients had higher proportions of Tumor Mutational Burden (TMB) and Microsatellite Instability (MSI) (Figure 8D). TMB, MSI, and mRNAsi scores were significantly higher in the high-risk group (Figure 8E). Survival analysis indicated that high TMB and MSI scores were associated with poorer OS (Figure 8F). Additionally, survival analysis combining risk score with TMB and MSI revealed that patients in the low TMB + low-risk score group had better OS than those in the high TMB + high-risk score group (P < 0.001), and the low MSI + low-risk score group had better OS than the high MSI + high-risk score group (P < 0.001) (Figure 8G).

### Immune cell infiltration analysis

**FIGURE 9:**
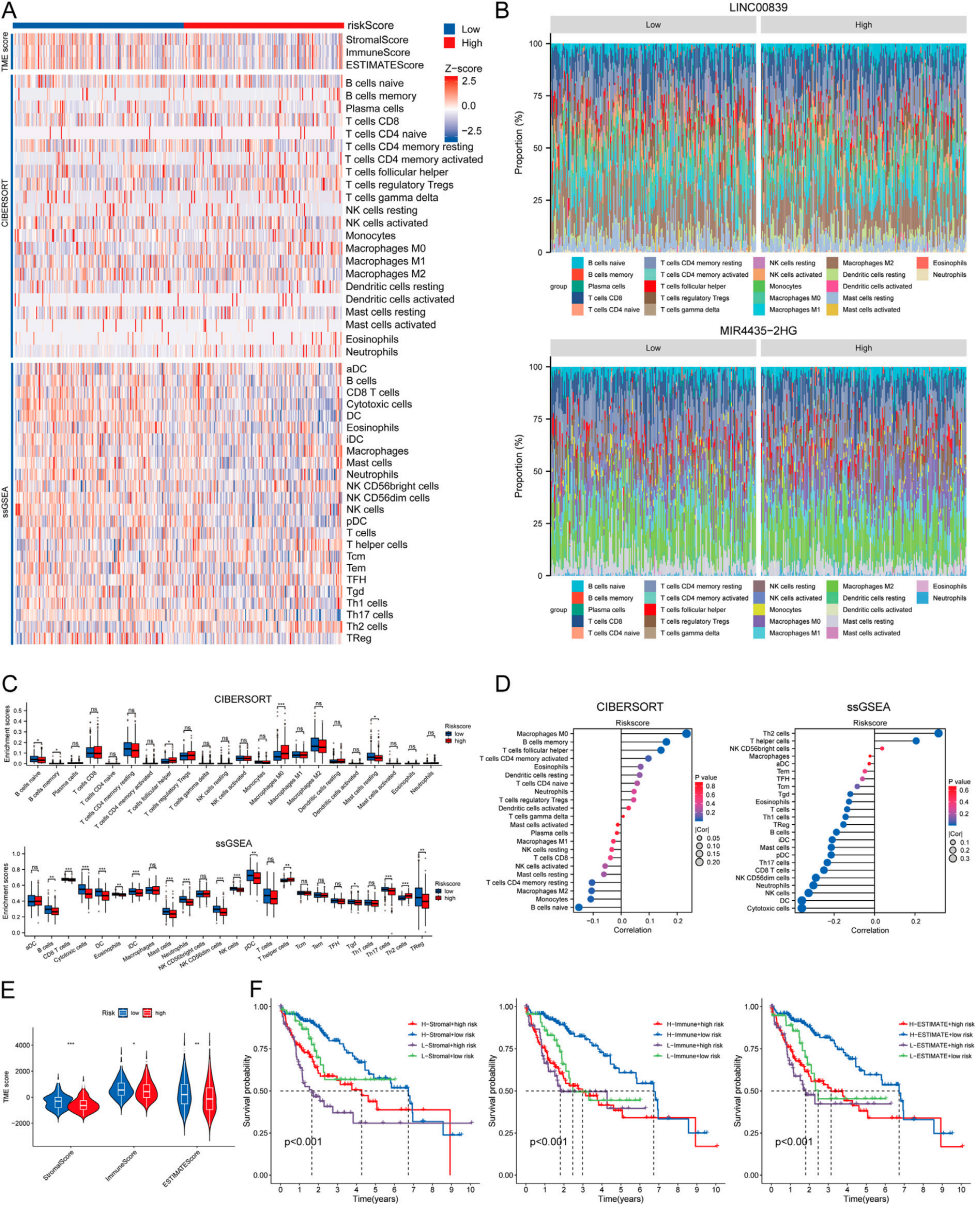
Relationship Between Risk Score and Immune Infiltration in the Tumor Microenvironment. **(A)** Heatmap of immune cell scores between high and low-risk score groups using CIBERSORT, ssGSEA, and ESTIMATE algorithms; **(B)** Percent abundance of tumor-infiltrating immune cells between high and low expression groups of two prognostic MRlncRNAs, with different colors representing various immune cell types; **(C)** Comparison of immune scores between high and low-risk score groups using CIBERSORT and ssGSEA; **(D)** Correlation analysis between risk score and immune infiltration using CIBERSORT and ssGSEA; **(E)** Differences in ESTIMATE between high and low-risk score groups; **(F)** Kaplan-Meier curves of four groups classified by risk score and three ESTIMATE scores in LIHC.

Using CIBERSORT, ssGSEA, and ESTIMATE algorithms, the correlation between risk score and immune cell infiltration was analyzed. Immune infiltration scores showed marked differences between high- and low-risk MRlncRNA groups (Figure 9A). The stacked bar chart depicted the immune cell infiltration proportions between high and low expression groups (Figure 9B). The CIBERSORT algorithm revealed that naive B cells, memory B cells, and resting mast cells were more abundant in the low-risk group, while follicular helper T cells and M0 macrophages were more abundant in the high-risk group. Risk score was positively correlated with eosinophils, activated CD4 T cells, follicular helper T cells, memory B cells, and M0 macrophages, and negatively correlated with naive B cells, monocytes, M2 macrophages, resting CD4 T cells, resting mast cells, activated NK cells, CD8 T cells, resting NK cells, and M1 macrophages. Additionally, ssGSEA analysis showed higher expression of B cells, CD8 T cells, cytotoxic cells, dendritic cells (DC), eosinophils, immature DC, mast cells, neutrophils, NK CD56dim cells, NK cells, plasmacytoid DC (pDC), Tγδ cells, and regulatory T cells (TReg) in the low-risk group, while T helper cells and Th2 cells were more expressed in the high-risk group (Figures 9C,D). Correlation analysis also indicated that risk score was negatively correlated with immune scores (Figure 9E). Prognostic analysis revealed that patients in the low ESTIMATE + high-risk score group had worse OS than those in the high ESTIMATE + low-risk score group (P < 0.001) (Figure 9F).

### Single-cell RNA sequencing (ScRNA-seq) data analysis

**FIGURE 10:**
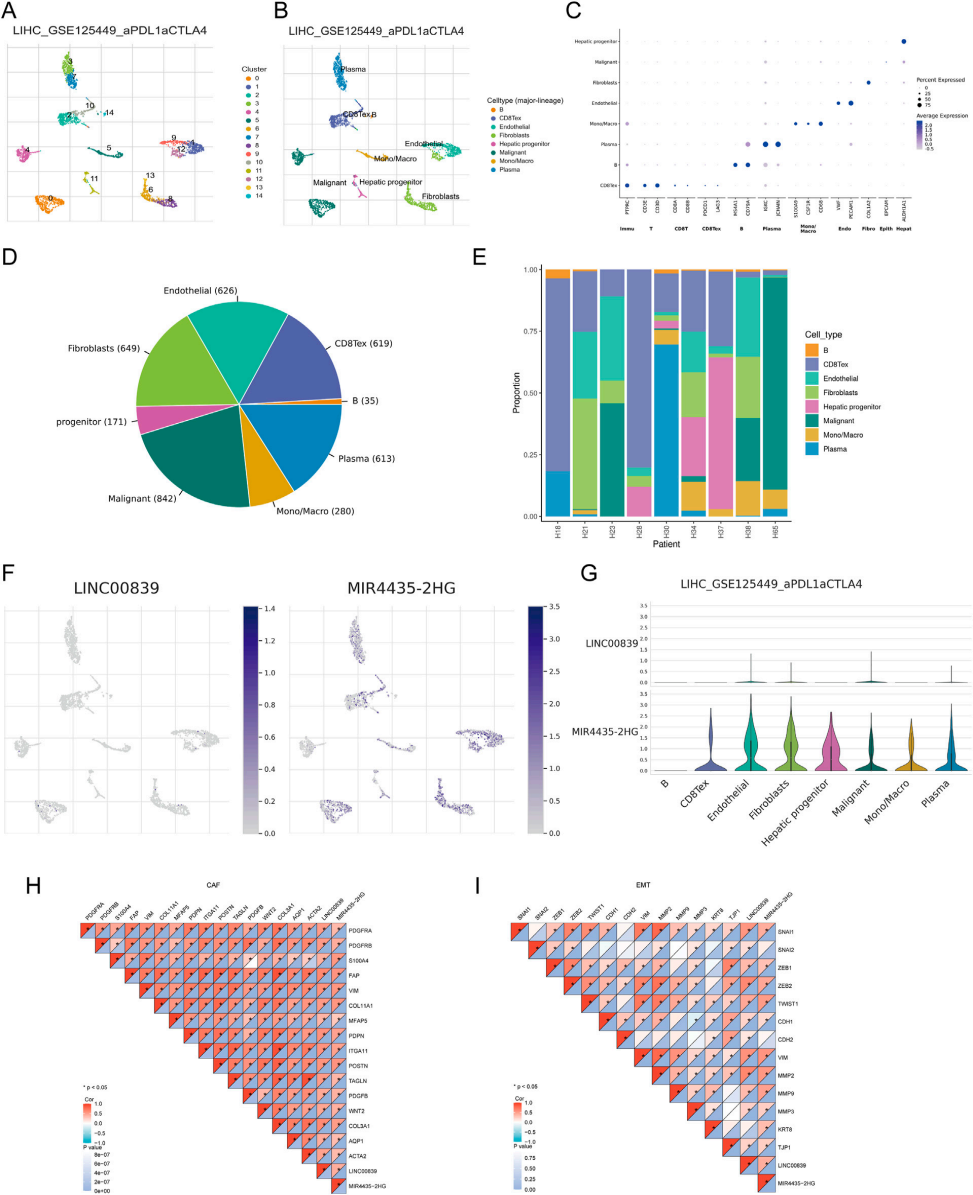
Expression of MRlncRNAs in Different Immune Cell Types in LIHC. **(A)** Clustering of cell types from scRNA-seq data; **(B)** Annotation of different immune cell types in LIHC tissues (LIHC_GSE125449); **(C)** Expression levels of marker genes in each cell cluster; **(D)** Pie chart showing the percentage of each cell type; **(E)** Percentage of each cell subtype in different patients; **(F)** Expression distribution of LINC00839, MIR4435-2HG in various cell types using single-cell resolution from the LIHC_GSE125449 dataset; **(G)** Violin plots showing the expression of LINC00839 and MIR4435-2HG in LIHC cells; **(H,I)** Correlation between LINC00839, MIR4435-2HG and CAFs or EMT-related markers.

Analysis of ScRNA-seq data from the LIHC_GSE125449 dataset in the TISCH database identified 14 cell clusters and eight cell types, highlighting the distribution and abundance of various TME-related cells (Figures 10A,B). A bubble plot visualized the expression of key marker genes across different cell types (Figure 10C). The pie chart showed higher expression in malignant cells, fibroblasts, and endothelial cells (Figure 10D). The distribution of LINC00839 and MIR4435-2HG expression in different cell types revealed that MIR4435-2HG had higher infiltration in TME-related cells than LINC00839 (Figures 10F,G). Further analysis showed that LINC00839 and MIR4435-2HG were significantly associated with cancer-associated fibroblasts (CAFs) and epithelial-mesenchymal transition (EMT) markers (Figures 10H,I). These results suggest that MRlncRNAs may promote fibrosis activation and contribute to LIHC metastasis via EMT.

### Immunotherapy response analysis

**FIGURE 11:**
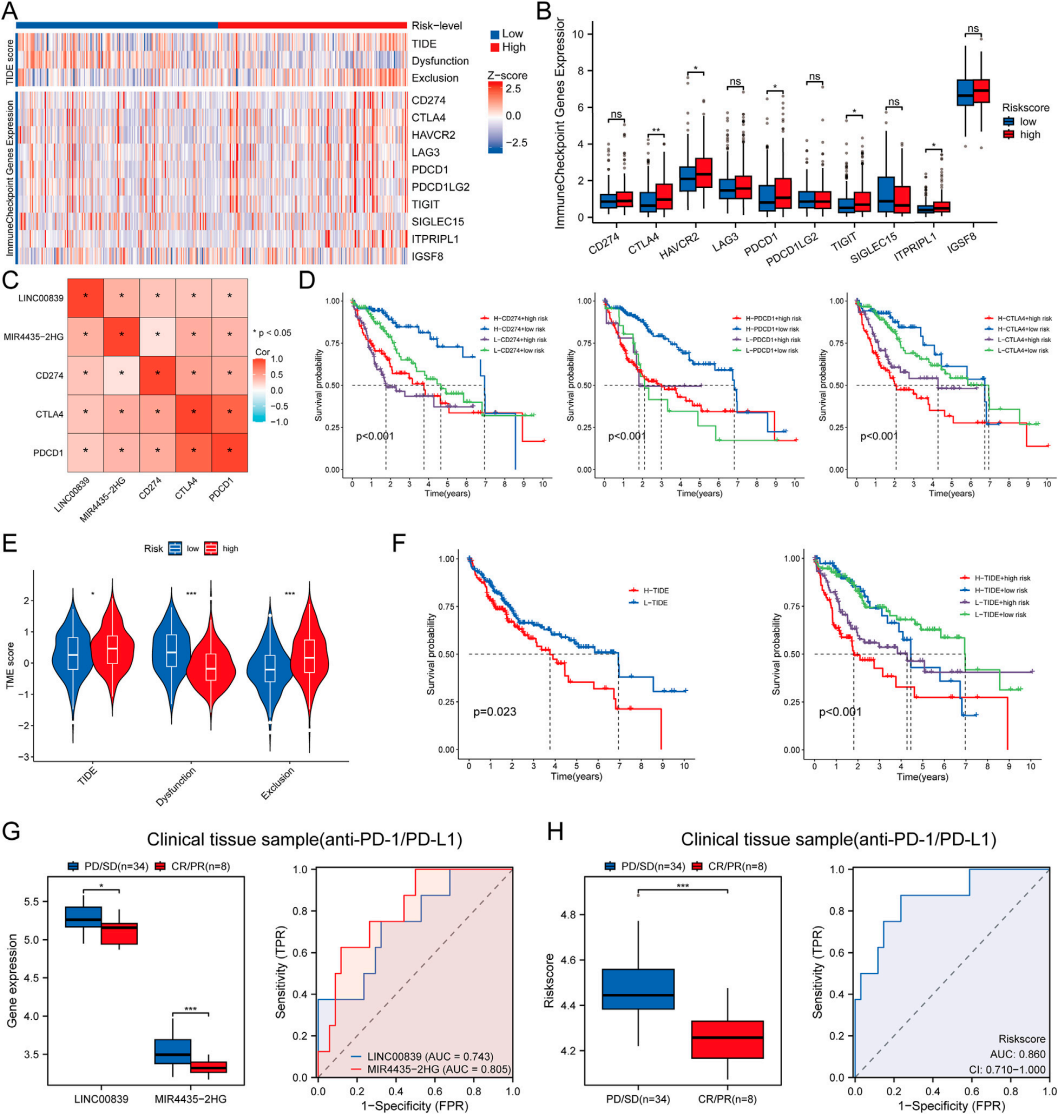
Immune Response Analysis. **(A)** Heatmap of immune checkpoint gene expression and TIDE scores between high and low-risk score groups, with different colors representing expression trends across different samples; **(B)** Expression distributions of eight immune checkpoint-associated genes between high and low-risk score groups in LIHC; **(C)** Correlation between risk score and clinical immune checkpoint-related genes in LIHC; **(D)** Kaplan-Meier survival curves for four groups classified by risk score and CD274, PDCD1, CTLA expression; **(E)** TIDE score evaluation of risk scores in response to immunotherapy in LIHC; **(F)** Kaplan-Meier survival plots of overall survival for high and low TIDE scores; **(G,H)** Differences in MRlncRNAs expression and risk score between patients with SD/PD and CR/PR in clinical tissue samples; ROC analysis of MRlncRNAs expression and risk score for predicting ICI responsiveness in clinical tissue samples (NR: non-responders; R: responders to immunotherapy).

Heatmap analysis showed significant differences in the distribution of immune checkpoint genes and TIDE scores between high- and low-risk groups (Figure 11A). Expression analysis of eight immune checkpoint-related genes revealed higher expression of CTLA4, HAVCR2, PDCD1, TIGIT, and ITPRIPL1 in the high-risk group (Figure 11B). The relationship between risk score and three key immune checkpoints (CD274, PDCD1, CTLA4) showed positive correlations (P < 0.05) (Figure 11C). Survival analysis indicated that patients with high immune checkpoint expression in the high-risk group had poorer OS compared to those with low checkpoint expression in the low-risk group (Figure 11D). To predict immune treatment response, TIDE scores and clinical sample data were used. Compared to the low-risk group, the high-risk group showed higher TIDE and Exclusion scores, and lower Dysfunction scores (Figure 11E). Kaplan-Meier analysis showed that the high-risk and high-TIDE group had the worst prognosis (Figure 11F). In an external clinical cohort of 80 liver cancer samples, patients in the low-risk group had higher rates of complete or partial remission (CR/PR) with immune checkpoint inhibitors (AUC >0.7) (Figures 11G,H). These findings suggest that low-risk patients are more likely to benefit from immune checkpoint inhibitor (ICI) therapy, showing better post-treatment survival rates.

### Chemotherapy drug sensitivity

Chemotherapy drug sensitivity analysis for low-risk and high-risk groups revealed that the high-risk group had significantly lower IC50 values for multiple drugs, including 5-Fluorouracil, Doxorubicin, and Paclitaxel (Supplementary Figure S1). These results suggest that these drugs may be effective for treating high-risk LIHC patients.

### Cell and clinical sample validation

**FIGURE 12:**
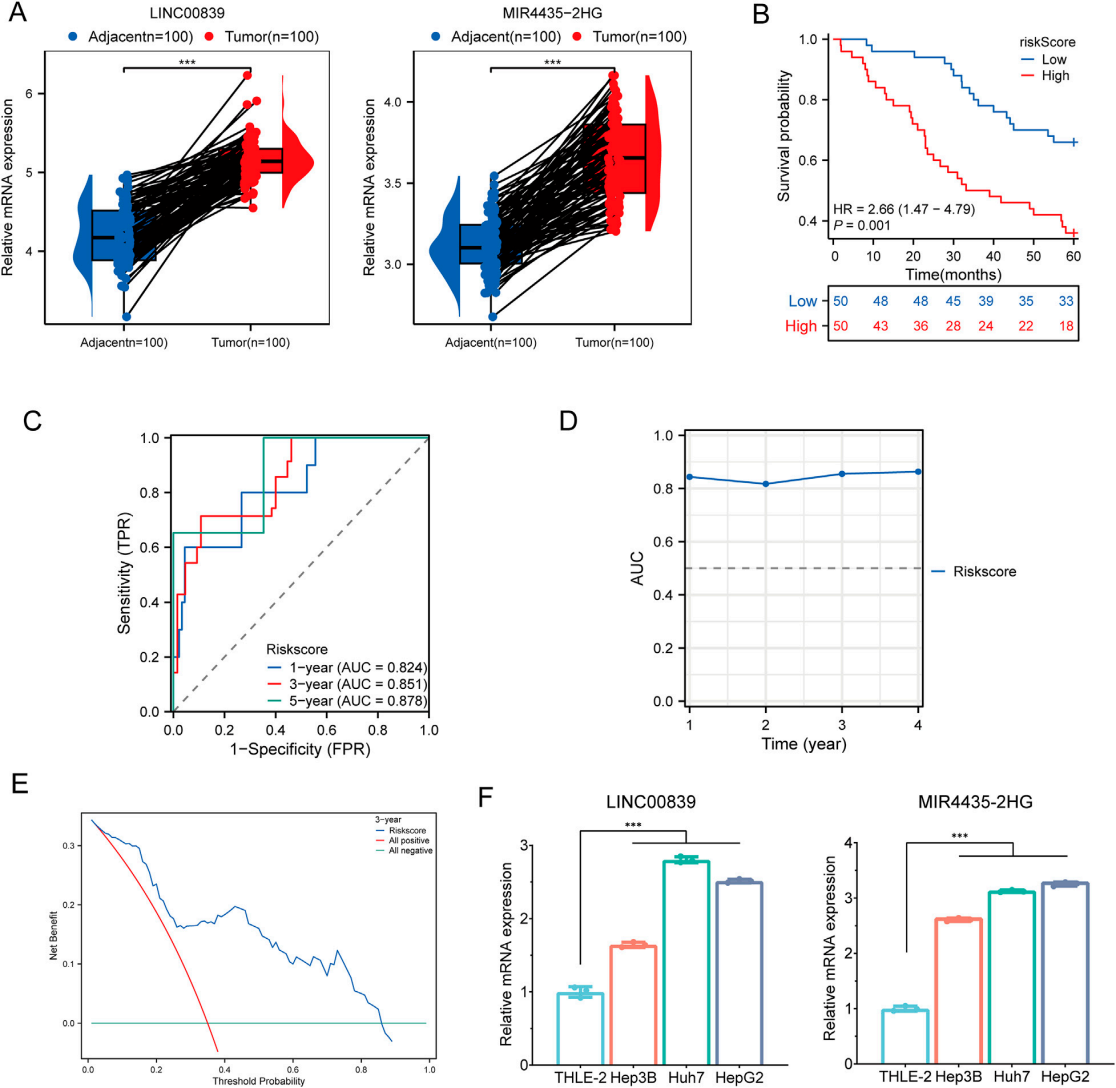
Clinical Sample and Cell Experiment Validation. **(A)** Relative expression of MRlncRNAs in normal and glioma tissues; **(B)** Overall survival curve for high- and low-risk HCC patients; **(C)** Time-dependent ROC curves for MRlncRNAs at 1, 3, and 5 years; **(D)** Time-dependent AUC curves; **(E)** Decision curve analysis for 3-year OS in clinical samples; **(F)** Differential expression of MRlncRNAs in HCC cell lines and normal cell lines.

To validate the expression and prognostic value of MRlncRNAs, we first performed RT-qPCR analysis in 100 paired hepatocellular carcinoma (HCC) and adjacent normal tissue samples. Both LINC00839 and MIR4435-2HG were significantly upregulated in tumor tissues (Figure 12A). Using the same cohort, patients were stratified into high- and low-risk groups based on the MRlncRNA-based risk score. Kaplan-Meier analysis revealed that high-risk patients had significantly poorer overall survival (OS) compared to low-risk patients (HR = 2.66, 95% CI: 1.47–4.79, P = 0.001; Figure 12B). Time-dependent ROC analysis showed strong predictive performance, with AUCs of 0.824, 0.851, and 0.878 for 1-, 3-, and 5-year OS, respectively (Figure 12C), and consistent accuracy over time (Figure 12D). Decision curve analysis (DCA) further demonstrated the clinical utility of the risk model for survival prediction (Figure 12E). To assess the robustness of the model, we randomly divided the 100-sample cohort into two equal subsets (Validation Set one and Validation Set two; each n = 50). In both subsets, the risk score effectively distinguished high-risk patients with significantly shorter OS (Supplementary Figure S2A, D). ROC curves yielded high AUCs in both sets: 0.833, 0.860, and 0.872 for Validation Set 1 (Supplementary Figure S2B); and 0.844, 0.855, and 0.856 for Validation Set 2 (Supplementary Figure S2E). DCA confirmed the model’s predictive benefit across both subsets (Supplementary Figure S2C, F, H). Moreover, RT-qPCR analysis in HCC cell lines (Hep3B, Huh7, and HepG2) and the normal hepatocyte line (THLE-2) confirmed significant overexpression of both MRlncRNAs in tumor cells, consistent with the clinical findings (Figure 12F). Together, these results validate the MRlncRNA-based risk model across bulk tissue samples and cell lines, underscoring its translational potential for clinical prognostication in HCC.

### MIR4435-2HG knockdown suppresses proliferation, migration, invasion, EMT, and PD-L1 expression in HCC cells

**FIGURE 13:**
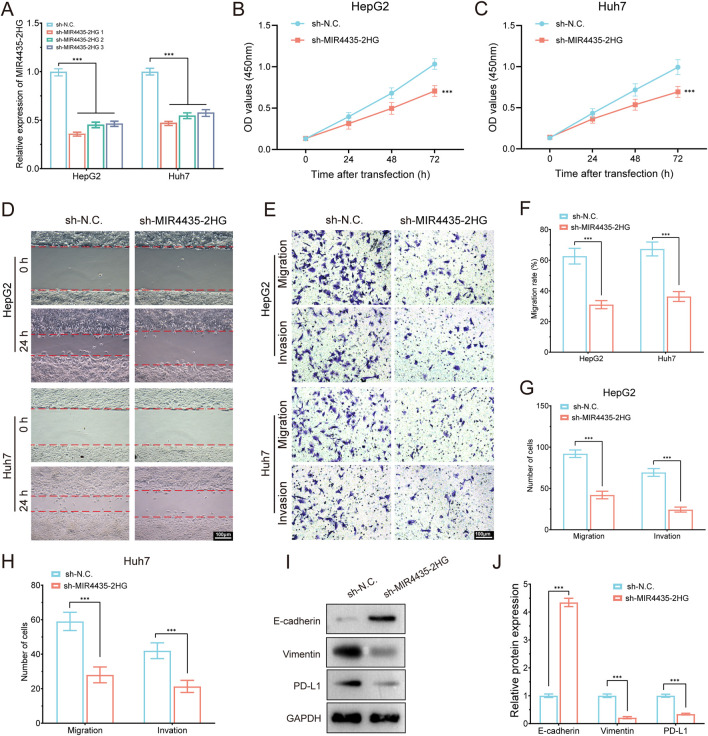
MIR4435-2HG knockdown suppresses proliferation, migration, invasion, EMT, and PD-L1 expression in HCC cells. **(A)** qRT-PCR showing knockdown efficiency of MIR4435-2HG in HepG2 and Huh7 cells using three shRNAs. **(B,C)** CCK-8 assays showing that MIR4435-2HG knockdown significantly inhibited cell proliferation in HepG2 **(B)** and Huh7 **(C)** cells at 24, 48, and 72 h. **(D)** Wound healing assay revealed impaired migration at 24 h in sh-MIR4435-2HG cells. **(E)** Transwell assays showed decreased migration and invasion. **(F–H)** Quantification of migration rates and cell numbers in HepG2 and Huh7. **(I)** Western blot showed increased E-cadherin and decreased Vimentin and PD-L1 upon MIR4435-2HG knockdown. GAPDH served as internal control. **(J)** Densitometric analysis of protein expression. Data are presented as mean ± SD; ***P < 0.001.

To validate the oncogenic function of MIR4435-2HG in hepatocellular carcinoma, we conducted a series of *in vitro* experiments in HepG2 and Huh7 cells. qRT-PCR confirmed efficient knockdown of MIR4435-2HG using three independent shRNAs, among which sh-MIR4435-2HG one exhibited the highest silencing efficiency and was selected for subsequent functional assays (Figure 13A). Cell proliferation was significantly inhibited upon MIR4435-2HG knockdown, as demonstrated by CCK-8 assays at 24, 48, and 72 h (Figures 13B,C). Wound healing assays revealed that MIR4435-2HG silencing impaired cell migration capacity at 24 h (Figures 13D,F). In addition, Transwell assays showed a marked reduction in both migratory and invasive abilities of HepG2 and Huh7 cells (Figures 13E,G,H). At the molecular level, Western blot analysis showed that MIR4435-2HG knockdown induced a mesenchymal–epithelial transition (MET)-like phenotype, as evidenced by upregulation of E-cadherin and downregulation of Vimentin (Figures 13I,J). Notably, the immune checkpoint molecule PD-L1 was also significantly downregulated in MIR4435-2HG–silenced cells, suggesting a potential role of this lncRNA in immune evasion. Together, these findings demonstrate that MIR4435-2HG promotes malignant phenotypes and immune escape in HCC, supporting its functional relevance in the migrasome-related lncRNA signature.

## Discussion

HCC remains a highly aggressive malignancy with poor prognosis, largely due to its molecular heterogeneity and immunosuppressive TME, which limit the efficacy of conventional and immune-based therapies. Migrasomes—recently identified as migration-dependent extracellular vesicles—have been implicated in tumor progression and immune modulation by transporting factors such as PD-L1 and TGF-β (Jiang et al., 2023; Deng et al., 2024). However, their interaction with lncRNAs, especially in the context of HCC, remains poorly understood. To address this gap, we conducted a systematic analysis of MRlncRNAs, aiming to uncover novel prognostic biomarkers and immune modulators.

By integrating transcriptomic and clinical data from TCGA-LIHC, we identified 191 candidate MRlncRNAs via Pearson correlation with 12 curated migrasome-related genes (|R| > 0.55, P < 0.001), of which 16 were associated with survival. LASSO Cox regression revealed a robust 2-lncRNA prognostic signature—LINC00839 and MIR4435-2HG—that minimized cross-validation error. This signature showed strong predictive accuracy (5-year AUC = 0.701 in TCGA, 0.872 in external cohort), indicating its broad applicability. RT-qPCR confirmed upregulation of both lncRNAs in HCC tissues and cell lines, using THLE-2 as the control, supporting the clinical relevance of our model. To elucidate functional relevance, we performed knockdown experiments targeting MIR4435-2HG in HepG2 and Huh7 cells. Silencing significantly suppressed proliferation, migration, and invasion, while increasing E-cadherin and decreasing Vimentin and PD-L1 expression, suggesting EMT reversal and reduced immune escape. These findings establish MIR4435-2HG as a key driver of both tumor aggressiveness and immunosuppressive remodeling. Additionally, LINC00839 is known to promote migration via hypoxia-induced FMNL2 activation (Xie et al., 2022), further linking MRlncRNAs to migrasome biogenesis and adaptive TME regulation. Collectively, our data support a model in which MRlncRNAs orchestrate EMT, immune checkpoint expression, and migrasome-related communication to drive HCC progression and immune evasion.

Our study revealed a strong association between high MRlncRNA expression and an immunosuppressive TME in HCC. Specifically, patients in the high-risk group exhibited elevated infiltration of M2 macrophages and regulatory T cells (Tregs), whereas low-risk patients showed enrichment of cytotoxic T lymphocytes and dendritic cells, indicating more active anti-tumor immunity (Guo et al., 2021; Song et al., 2025). These shifts suggest that MRlncRNAs contribute to immune evasion by shaping the immune landscape. Mechanistically, knockdown of MIR4435-2HG significantly reduced PD-L1 expression and suppressed EMT markers, directly linking MRlncRNAs to both immune checkpoint activation and tumor cell plasticity. M2 macrophages and Tregs—abundant in high-risk patients—are known to secrete IL-10 and TGF-β, which promote immune suppression and EMT (Kalluri, 2016). This supports a CAF–EMT–MRlncRNA regulatory axis that orchestrates immune exclusion, stromal remodeling, and PD-L1–mediated T cell inhibition (Wu et al., 2021). Further analysis revealed significantly higher expression of immune checkpoints (PDCD1, CTLA4, TIGIT, CD274) in high-risk patients. Integration of the MRlncRNA risk score with the TIDE algorithm markedly improved prediction of immunotherapy responsiveness, surpassing existing lncRNA-based models (Qin et al., 2023a). As immune checkpoint inhibitors (ICIs) become the frontline treatment for advanced HCC, accurate biomarkers such as MRlncRNA signatures are urgently needed to stratify patients and predict immunotherapy response, particularly given the complex clinical contexts such as liver transplantation, where ICIs pose potential risks of graft rejection (Sensi et al., 2024). While elevated TMB and MSI typically predict ICI benefit, high-risk patients in our cohort—despite high TMB/MSI—had poor outcomes, suggesting that MRlncRNA-driven immune suppression may offset these immunogenic features (Holder et al., 2024; Qi et al., 2024). This paradox highlights the complexity of the HCC microenvironment: heightened tumor immunogenicity may be neutralized by MRlncRNA-enhanced immune evasion. Collectively, our findings underscore the utility of MRlncRNAs as biomarkers of immune escape and resistance to immunotherapy in HCC.

Beyond their prognostic relevance, MRlncRNAs are functionally linked to the migrasome–CAF axis, a novel pathway contributing to tumor–stromal crosstalk and immune evasion in HCC. Migrasomes are recently identified migration-dependent extracellular vesicles involved in intercellular communication and immune regulation (Qiao et al., 2023). Several MRGs used in our co-expression analysis—TSPAN4, NDST1, and ITGAV—have been experimentally validated as essential for migrasome biogenesis and matrix interactions. Emerging studies show that HCC-derived migrasomes can carry immunosuppressive factors such as PD-L1 and TGF-β, fostering Treg differentiation and immune escape (Qin et al., 2023b). Although we did not directly localize MRlncRNAs within migrasomes, bioinformatic co-expression and functional associations strongly suggest a mechanistic interplay. MIR4435-2HG, for instance, is highly enriched in CAFs, as revealed by our scRNA-seq analysis (Cui et al., 2023). CAFs are key stromal components known to secrete TGF-β and IL-6, remodel the ECM, and promote immune suppression and tumor invasion (Qin et al., 2025). Moreover, MIR4435-2HG has been shown to regulate B3GNT5, a glycosyltransferase implicated in vesicle-mediated invasiveness (Qin et al., 2024). These findings support the hypothesis that MRlncRNAs—especially MIR4435-2HG—may influence migrasome biogenesis or cargo selection and potentiate CAF-driven immunosuppressive remodeling of the tumor microenvironment. This highlights a unique stromal regulatory circuit in HCC and suggests that MRlncRNAs may serve not only as prognostic biomarkers but also as therapeutic targets for disrupting the CAF–migrasome–immunity axis.

In addition to immune modulation, MRlncRNAs appear to orchestrate several tumor-promoting processes including EMT, metabolic reprogramming, and hypoxia adaptation. Our study revealed that MRlncRNAs are closely associated with EMT markers, supporting their role in promoting tumor invasiveness and metastasis in HCC. LINC00839 has been reported to upregulate SNAI2 and ZEB1, while MIR4435-2HG enhances expression of mesenchymal markers like Vimentin and N-cadherin, contributing to EMT activation (Xie et al., 2022; Guo et al., 2021). Notably, EMT also facilitates immune escape through PD-L1 upregulation, further implicating MRlncRNAs in immunosuppressive remodeling (Zhang et al., 2023). Moreover, MRlncRNAs may participate in cancer metabolic rewiring (Sun et al., 2022; Ghosh et al., 2021). Our enrichment analysis revealed associations with lipid metabolism and bile acid biosynthesis pathways, which are known to support tumor growth and immune evasion (Watterson and Coelho, 2023). Notably, many of the pathways associated with MRlncRNAs—including Wnt/β-catenin, mTOR, and MAPK—are well-established drivers of HCC progression and therapeutic resistance, as highlighted in recent reviews (Altaf et al., 2022). In particular, MIR4435-2HG may promote lipid metabolism, enabling HCC cells to thrive in nutrient- or oxygen-deprived conditions (Guo et al., 2021). This metabolic advantage may synergize with immune escape mechanisms to foster a more aggressive tumor phenotype. Hypoxia, a hallmark of advanced HCC, further amplifies these effects. We found that LINC00839 is upregulated under hypoxic conditions, promoting migration and invasion (Xie et al., 2022). This is consistent with earlier studies indicating that hypoxia-induced lncRNAs facilitate tumor adaptation and therapy resistance (Giraud et al., 2021; Pu et al., 2024). Therefore, MRlncRNAs may integrate hypoxia signaling, EMT progression, and metabolic alterations into a unified network that sustains tumor aggressiveness and immune evasion.

Our findings have several important clinical implications. First, the MRlncRNA-based risk score model provides a robust tool for prognostic stratification in HCC patients, enabling more personalized treatment strategies. Second, the association between MRlncRNAs and immune cell infiltration suggests that these lncRNAs may serve as therapeutic targets for enhancing anti-tumor immunity. For example, targeting MRlncRNAs with RNA interference (RNAi) or antisense oligonucleotides (ASOs) could potentially reverse the immunosuppressive TME and improve responses to ICIs. Future studies should focus on validating these findings in larger, multi-center cohorts and exploring the therapeutic potential of targeting MRlncRNAs in preclinical models. Additionally, the role of MRlncRNAs in other cancer types should be investigated to determine whether their functions are conserved across different malignancies. Finally, the development of MRlncRNA-targeted therapies will require careful consideration of delivery methods to ensure specificity and minimize off-target effects.

Despite the promising findings, this study has several limitations. First, both the TCGA-LIHC dataset and our external validation cohort (n = 100) were retrospectively collected, which may introduce selection bias. To address this, we expanded the clinical cohort and performed internal validation by dividing it into two independent subsets, each demonstrating robust and consistent prognostic performance. Nonetheless, future studies using prospectively collected, multi-center cohorts are essential to validate the generalizability of our MRlncRNA-based model. Second, while previous studies on lncRNAs in HCC have focused on canonical oncogenic molecules like HOTAIR or MALAT1, our work is the first to systematically characterize MRlncRNAs in this context. Through integrated multi-omics approaches—encompassing transcriptomic screening, prognostic modeling, clinical validation, and scRNA-seq—we uncovered a novel CAF–EMT–immune checkpoint regulatory axis potentially modulated by migrasome signaling. Importantly, we provided direct experimental support: silencing MIR4435-2HG impaired tumor proliferation, migration, invasion, and reversed EMT and immune evasion phenotypes (upregulation of E-cadherin; downregulation of Vimentin and PD-L1). Finally, our 2-lncRNA signature (LINC00839 and MIR4435-2HG) demonstrated strong prognostic power and mechanistic relevance. Its integration with the TIDE algorithm significantly enhanced the prediction of immunotherapy response, outperforming existing lncRNA-based models. In contrast to models focused solely on tumor-intrinsic features, our signature reflects both metabolic reprogramming and immune exclusion. These findings highlight the translational potential of MRlncRNAs—and by extension, the migrasome–CAF–immune checkpoint axis—as promising therapeutic targets in HCC.

## Conclusion

In summary, we developed and validated a novel 2-lncRNA signature (LINC00839 and MIR4435-2HG) associated with tumor progression and immune suppression in HCC. Using 100 clinical samples and functional assays, we confirmed that MIR4435-2HG promotes EMT and PD-L1–mediated immune evasion. Our integrative model, combining MRlncRNA expression with the TIDE algorithm, enhances immunotherapy response prediction beyond existing signatures. Moreover, scRNA-seq and *in vitro* data support a potential CAF–EMT–immune checkpoint axis driven by MRlncRNAs. These findings highlight the translational potential of targeting migrasome-related lncRNAs for improving prognostic assessment and immunotherapy outcomes in HCC.

## Data Availability

The datasets are available in TCGA database (https://portal.gdc.cancer.gov/), GeneCards 586 (https://www.genecards.org/). Further inquiries can be directed to the corresponding authors.
