# Supplementary material for: A migrasome-related lncRNA signature predicts prognosis and immune response in hepatocellular carcinoma: Implications for biomarker discovery and therapeutic targeting
Source: Front Pharmacol. 2025 Aug 6;16:1581122. doi: 10.3389/fphar.2025.1581122 (PMC12364852; doi:10.3389/fphar.2025.1581122)
Supplement: Supplementary file 1 [file DataSheet1.zip › Supplementary Table 4.docx]

| Characteristics | low-risk | high-risk | P value |
| --- | --- | --- | --- |
| n | 191 | 179 |  |
| Age, n (%) |  |  | 0.051 |
| <=60 | 82 (22.2%) | 95 (25.7%) |  |
| >60 | 109 (29.5%) | 84 (22.7%) |  |
| Gender, n (%) |  |  | 0.905 |
| FEMALE | 63 (17%) | 58 (15.7%) |  |
| MALE | 128 (34.6%) | 121 (32.7%) |  |
| Grade, n (%) |  |  | < 0.001 |
| G1 | 41 (11.2%) | 14 (3.8%) |  |
| G2 | 98 (26.8%) | 79 (21.6%) |  |
| G3 | 46 (12.6%) | 75 (20.5%) |  |
| G4 | 3 (0.8%) | 9 (2.5%) |  |
| Stage, n (%) |  |  | 0.009 |
| Stage I | 103 (29.8%) | 68 (19.7%) |  |
| Stage III | 40 (11.6%) | 45 (13%) |  |
| Stage II | 33 (9.5%) | 52 (15%) |  |
| Stage IV | 2 (0.6%) | 3 (0.9%) |  |
| T, n (%) |  |  | 0.002 |
| T1 | 109 (29.7%) | 72 (19.6%) |  |
| T4 | 8 (2.2%) | 5 (1.4%) |  |
| T3 | 37 (10.1%) | 43 (11.7%) |  |
| T2 | 34 (9.3%) | 59 (16.1%) |  |
| M, n (%) |  |  | 0.307 |
| M0 | 135 (50%) | 131 (48.5%) |  |
| M1 | 1 (0.4%) | 3 (1.1%) |  |
| N, n (%) |  |  | 0.299 |
| N0 | 129 (50.4%) | 123 (48%) |  |
| N1 | 1 (0.4%) | 3 (1.2%) |  |
