# Supplementary material for: A migrasome-related lncRNA signature predicts prognosis and immune response in hepatocellular carcinoma: Implications for biomarker discovery and therapeutic targeting
Source: Front Pharmacol. 2025 Aug 6;16:1581122. doi: 10.3389/fphar.2025.1581122 (PMC12364852; doi:10.3389/fphar.2025.1581122)
Supplement: Supplementary file 1 [file DataSheet1.zip › Supplementary Figures.docx]

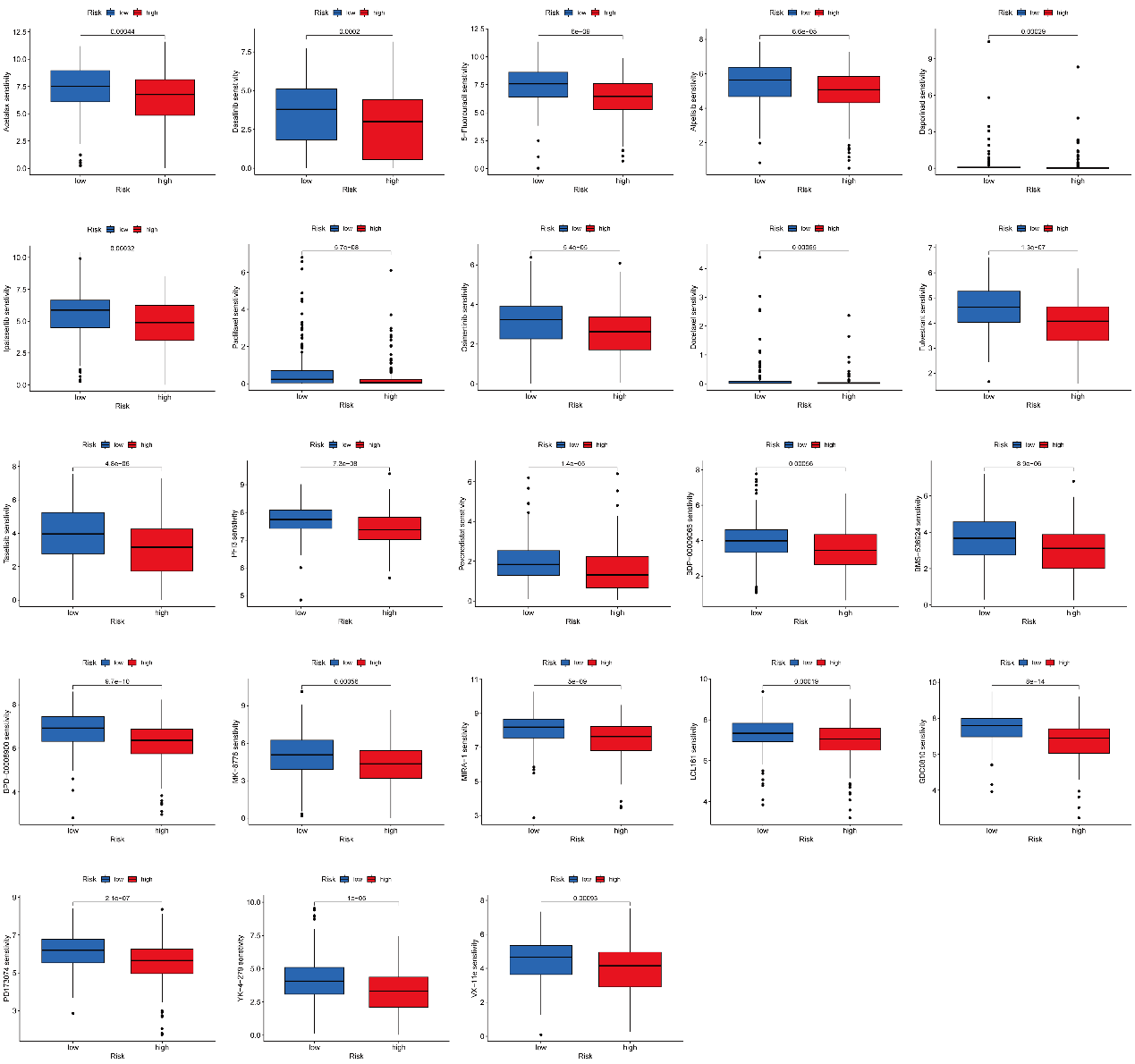


**Figure S1:** Chemotherapy Drug Sensitivity Analysis.


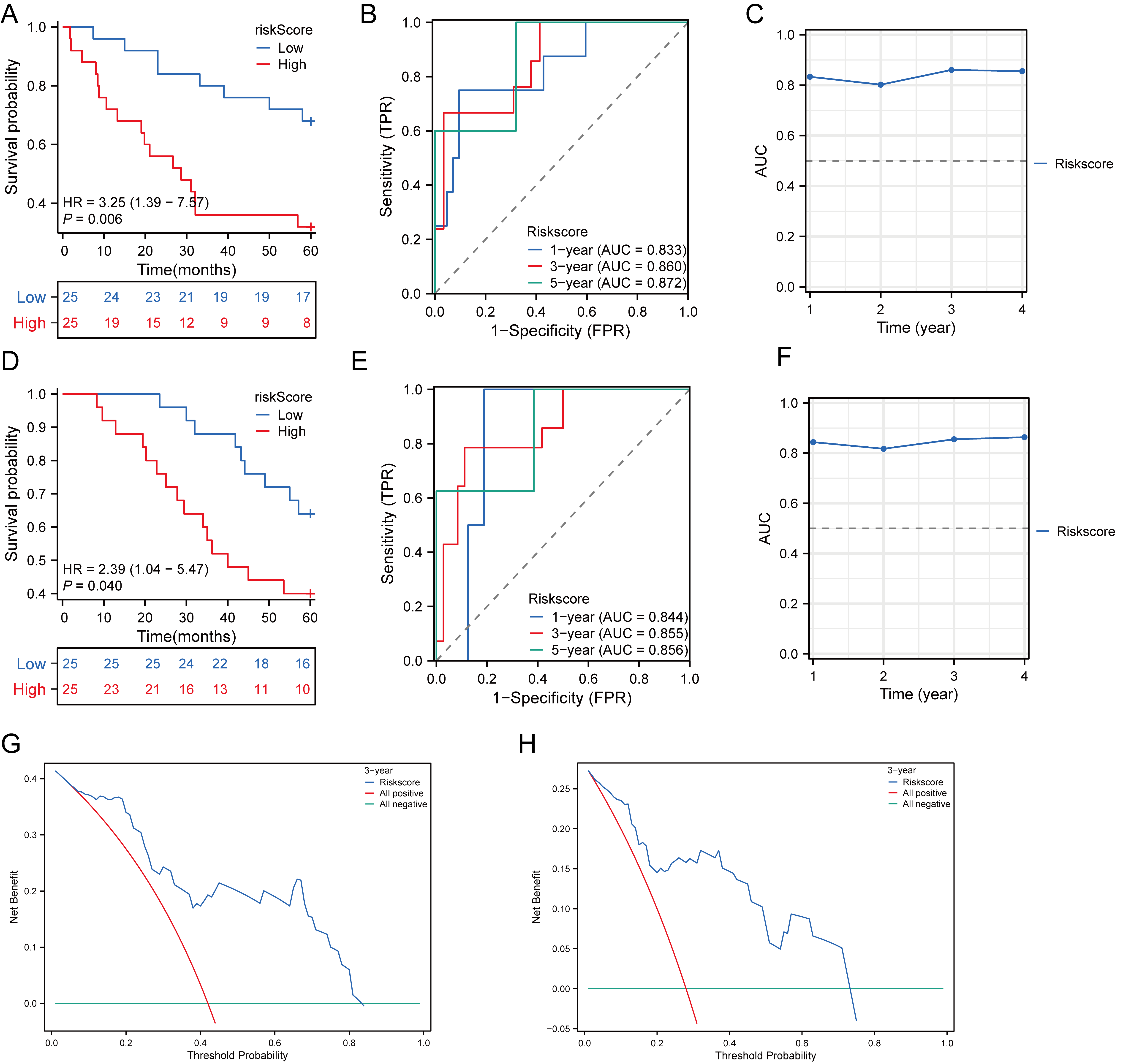
**Figure S2:** Prognostic value of MRGs signature and risk score validation in clinical samples. (A) Risk score and survival probabilities in clinical tissue validation set 1; (B) Time-dependent ROC curves for MRlncRNAs at 1, 3, and 5 years in clinical tissue validation set 1; (C) Time-dependent AUC curves in clinical tissue validation set 1; (D) Risk score and survival probabilities in clinical tissue validation set 2; (E) Time-dependent ROC curve analyses of risk score in clinical tissue validation set 2; (F) Time-dependent ROC curve analyses of risk score in clinical tissue validation set 2; (G, H) Decision curve analysis for 3-year OS in clinical tissue validation set 1,2.
